# Relative density of United States forests has shifted to higher levels over last two decades with important implications for future dynamics

**DOI:** 10.1038/s41598-021-98244-w

**Published:** 2021-09-22

**Authors:** C. W. Woodall, A. R. Weiskittel

**Affiliations:** 1 USDA Forest Service, Northern Research Station, 271 Mast Rd, Durham, NH USA; 2 Center for Research On Sustainable Forests, University of Maine, 5755 Nutting Hall, Orono, ME 04469-5755 USA

**Keywords:** Forestry, Forest ecology, Forest ecology

## Abstract

Tree size-density dynamics can inform key trends in forest productivity along with opportunities to increase ecosystem resiliency. Here, we employ a novel approach to estimate the relative density (RD, range 0–1) of any given forest based on its current size-density relationship compared to a hypothetical maximum using the coterminous US national forest inventory between 1999 and 2020. The analysis suggests a static forest land area in the US with less tree abundance but greatly increased timber volume and tree biomass. Coupled with these resource trends, an increase in RD was identified with 90% of US forest land now reaching a biologically-relevant threshold of canopy closure and/or self-thinning induced mortality (RD > 0.3), particularly in areas prone to future drought conditions (e.g., West Coast). Notably, the area of high RD stands (RD > 0.6) has quintupled over the past 20 years while the least stocked stands (RD < 0.3) have decreased 3%. The evidence from the coterminous US forest RD distribution suggest opportunities to increase live tree stocking in understocked stands, while using density management to address tree mortality and resilience to disturbances in increasingly dense forests.

## Introduction

Forests continue to emerge as one of the key fulcrums of climate change both directly via provisioning of ecosystem services (e.g., clean air/water and net carbon sequestration^1,2^) and indirectly via feedback mechanisms such as increased tree mortality^3^ with subsequent CO_2_ emissions^4^ (e.g., decay and wildfires) that may accelerate climate change^5^. Compounding these dynamics, global trade and transportation involving forest products (e.g., packing materials or sawlogs) has increased the prevalence and impact of invasive species across forest ecosystems of North America^6^. As a result, fundamental metrics of forest attributes and dynamics (e.g., area, mortality, or biomass) across scales ranging from stands to nation/biome/continent are garnering increased examination^7^ in the context of global change policies addressing forest ecosystem restoration (e.g., Trillion Trees Initiative, 1t.org, www.reforestationhub.org), carbon sequestration potential^8^, and/or adaptive management opportunities that may increase forest resilience to future disturbances^9^. Metrics based on metabolic scaling theory may be particularly relevant to emerging regional and national forest resource questions^10^ as their formulation is based on fundamental plant size-density tenets and can be readily applied to basic forest inventory data^11^. Although there are numerous forest management metrics (e.g., annual net carbon sequestration) that can address the current and future status of forests, an evaluation of the current size-density attributes of US forests provides an opportunity to deepen discussions pertaining to strategic-scale tree planting and thinning activities that fundamentally must adhere to metabolic scaling theories that constrain tree size-density attributes of forests.

Live tree size-density metrics have informed the management of forest stands for many decades^12,13^. Although absolute metrics of forest stand attributes such as tree abundance and carbon stocks^14^ across national scales are vitally important for the development of forest policies, size-density metrics enable estimation of the current status of any given forest stand relative to a maximum size-density relationship. Importantly, such metrics are founded on the fundamental understanding of ecophysiology and production economics aspects^15^ which can be efficiently applied by land managers using the common stand parameters of tree species, size, and abundance (i.e., stand size-density management diagrams)^16^. Over the past decade, the emergence of digitally available forest inventories across large spatial scales^17^, functional trait science^18^, and available climate data have brought about a re-examination of size-density metrics^19^ as a means to interpret the density of live trees across spectrums of climates, tree species assemblages, and structurally complex stands that, we hypothesize, will co-mingle and shift over time due to global change. Coincident with this trend is the need to estimate stocking characteristics (i.e., current number and size of trees related to expected thresholds of self-thinning induced mortality or canopy closure) of novel tree species in changing climates using adaptive management techniques as a means to increase the resilience of future forests^9,20^. Size-density management diagrams^16^ for mono-specific stands for a specific region or type of site based on historic stand inventories are most likely unable to meet such needs, which may usher in a new era of stand density assessments that are optimized for their scale of implementation (nation, state, stand), while accommodating myriad species, site, and tree size combinations.

Of the most commonly implemented size-density metrics in forests is the stand density index^13,21^ (SDI; # ha^−1^). Reineke^12^ first proposed SDI as a mathematical solution to quantify the biological phenomena of a finite number of trees of a certain size (e.g., # of trees with a diameter of 25.4 cm per ha) that could occupy any given forest stand/site. For a given size of tree (no minimum diameter), the ecophysiological attributes of any tree species limit the number of trees that can occupy a given site with the frontier of these size and abundance relationships defining a maximum stand density^13^. Regardless of the formulation, the SDI of any stand, just like live tree volume or biomass, is an absolute measure of a forest stand’s current occupancy of “packing space”^15^. In order to estimate a relative density (RD) of trees for any given forest, a maximum potential SDI (i.e., theoretical maximum number of trees per unit area of a given size, SDI_max_) must be concomitantly estimated. Given the breadth of emerging literature on the topic of formulating both SDI and RD^19,22–26^ in the context of society’s need to objectively quantify the current state of live tree stocking across scales and diverse applications, a comprehensive, national-scale size-density assessment of US forests is needed and now possible given available data and analytical methods.

Although determination of RD for any given forest can provide an objective quantification as to where a forest lies in the continuum of size-density configurations (i.e., a few small trees versus many large trees per unit area), there remains subjectivity in related biological and policy interpretations. RD of forests can be evaluated across large scales, time, and in the context of future climates, but it is terms such as “stocking” of live trees that inherently assigns a subjective value to the current size-density status of any given forest. Therefore, for the purpose of evaluating RD results across US forests we classify forests according to three levels of RD related to widely accepted management/biological thresholds: (1) low (a density below full canopy closure thresholds), (2) medium (a density that achieves full canopy closure and/or exclusion of new individuals while minimizing self-thinning mortality), and (3) high (a density of imminent self-thinning mortality). Our hope is that such general classifications will enable inclusive discussions of current forest dynamics while highlighting needed areas for further research, especially in the context of expected future forest disturbances. In terms of disturbances, the increasing occurrence and severity of drought^27^ throughout the US^3^ suggests that intersecting high RD forest populations with drought projections may have important implications in terms of US forests and their susceptibility to major natural disturbances like fire or insect outbreaks.

Our goal here is to develop a robust, interpretable, and nationally consistent approach for quantifying the RD of live trees across time that is applicable to all forests of the coterminous US. Specific objectives are to: (1) develop a nationally comprehensive and temporally consistent approach for estimating the strategic-scale RD (i.e., proximity to self-thinning line) of live trees that incorporates varying species composition, stand structure, and site attributes using the US national forest inventory (sub-plot observations > 1 million), (2) evaluate potential spatial and temporal drivers of RD (e.g., forest type, ecoregions), (3) estimate decadal changes (1999–2012 to 2013–2020) in forest RD across the coterminous US by units of interest (e.g., states) against a context of changes in complementary US forest metrics (e.g., abundance and biomass), and (4) explore one RD application to a contemporary forest policy issue by summarizing current levels of US forest RD related to future drought severity indices.

## Results

**Figure 1:**
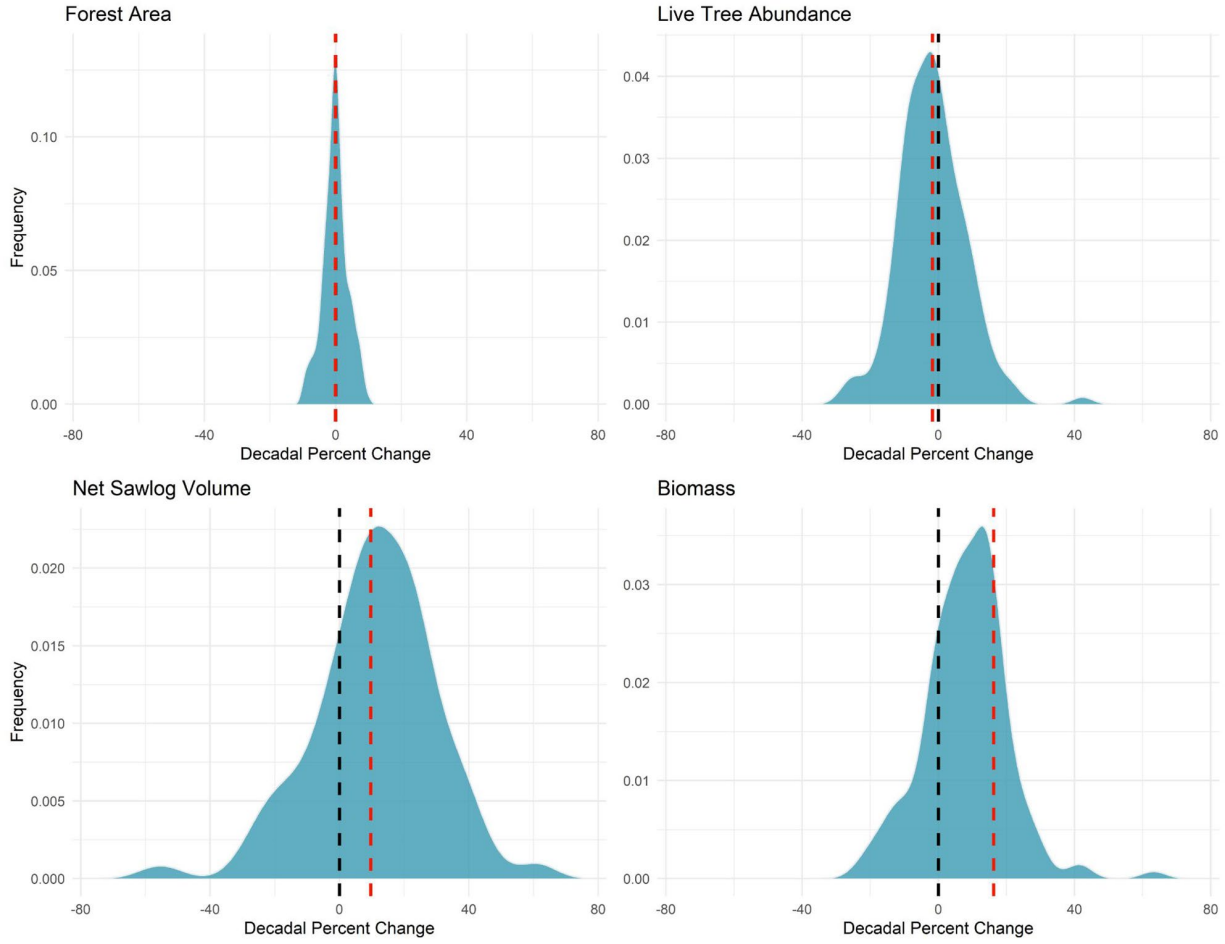
Percent change of forest attributes (forest land area, live tree abundance, net sawlog volume, and total live tree biomass) among coterminous US forest ecological sections, 1999–2012 to 2013–2020. Zero percent change indicated by dashed black line while mean percentage change indicated by dashed red line. *Note*: Ecological sections with minor amounts of forest that had potentially spurious decadal change ±10% removed from display.

A total of 1,257,773 sub-plot observations covering a wide range of coterminous US conditions were used for determining maximum SDI (SDI_max;_ see Supplementary Tables 1 & 2), with changes in the mean estimates of tree size, basal area, and abundance all indicating a general progression towards the self-thinning line as a whole (i.e., larger but fewer trees with greater live tree basal area at the stand-level). The determined intercept and slope of Eq. (1) were 12.634 and −1.797, respectively, with high variation in the plot-specific parameters and a mean implied SDI_max_ of 917.0. The standard deviation of the intercept (ʋ_i_) and slope random effects (ɣ_i_) were 0.119 and 0.106, respectively, with a strong correlation between estimates (r^2^ = 0.926). The distribution of percentage change in the key forest resource metrics of forest land area, live tree abundance, net sawlog volume, and live tree aboveground biomass among delineated ecosystems indicate that US forest area and tree abundance are relatively static, while live tree volume and total biomass continue to increase for the majority of observed ecosystems (Fig. 1).

Implied plot-level SDI_max_ exhibited a wide distribution of values with a range of 4426.5 and the highest observations occurring primarily in the western forests of California, Oregon, and Washington (see Supplementary Figure 1). Primary factors influencing variation in SDI_max_ were forest type, standard deviation of the diameter distribution, EPA Level III ecoregions, and climatic factors (see Supplementary Figure 3). Variables representing species composition and functional traits had a more limited influence on SDI_max_ at a national scale.

**Figure 2:**
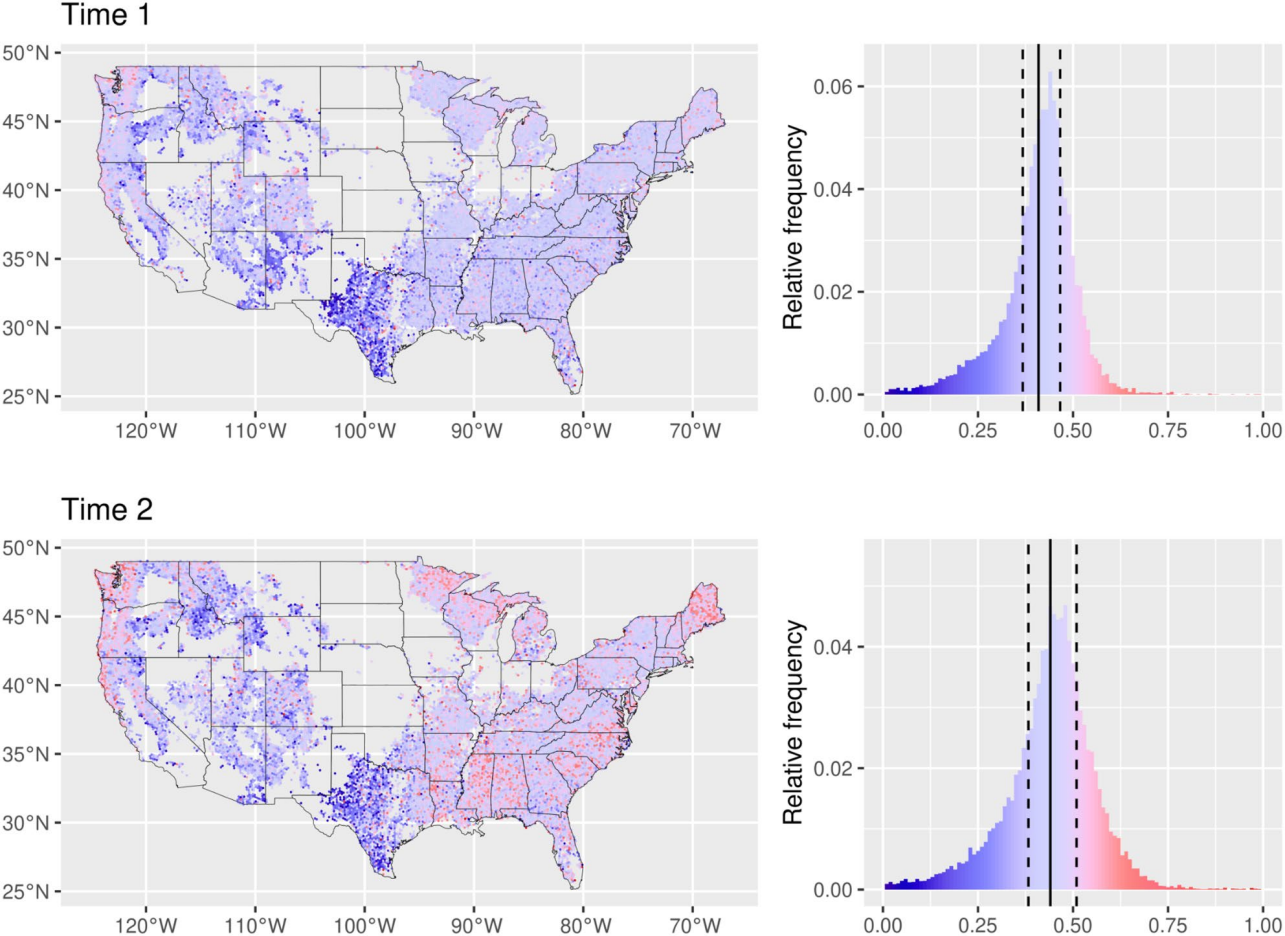
Observed spatial (left) and relative frequency (right) of mean relative density (RD) by study hexagon (277.3 km^2^) for Time 1 (1999–2012) and Time 2 (2013–2020). Dark blue colors reflect RD < 0.3, dark red colors reflect RD > 0.6, median (solid line) and 1st/3rd quartiles (dotted line) are noted in the relative frequency distribution. Map produced in R v3.6.3^52^.

A total of 208,517 plot-level observations were used in assessing the spatial and temporal variation in RD (Table 1). RDs below 0.3 are considered to be low (i.e., lack of canopy closure), RDs between 0.3 and 0.6 are considered to be medium, and RDs in excess of 0.6 are considered to be high (i.e., imminent tree mortality). In a qualitative sense, the terms “low,” “medium,” and “high” RD are intended to parsimoniously refer to the size-density status of forests (i.e., RD thresholds relevant to full canopy closure and self-thinning) and not to the stocking of a diverse array of ecosystem attributes/services. Similar to SDI_max_, the most influential factors on RD were forest type, standard deviation of the diameter distribution, and EPA Level III ecoregions, while ownership (private vs. Federal vs. state), climate, and other site factors had a rather limited influence. Although the Time 1 (1999-2012) mean RD is not statistically different from Time 2 (2013-2020; 0.42 ± 0.15 vs 0.46 ± 0.18), the overall distribution has skewed towards higher RD values at Time 2 (Fig. 2). Based on ecologically and forest management relevant thresholds^28^ (e.g., full canopy closure), 5.8% of the US forest area is now above the high RD value of 0.60 (onset of self-thinning) compared to 1.0% at Time 1 (see Supplementary Table 3).

**Table 1:** Plot-level (*N* = 208,517) summary of key structural and compositional attributes by Time 1 (1999–2012) and Time 2 (2013–2020).

Attribute^a^	Mean	SD	Minimum	Maximum
**Time 1 (1999–2012; N = 114,116)**				
TPH (# ha^−1^)	1438.6	1366.3	2.5	25,095.9
QMD (cm)	17.9	10.0	2.5	168.7
BAPH (m^2^ ha^−1^)	22.9	14.6	0.1	295.9
pHW.BA	68.4	34.9	0.0	100.0
SDI (# ha^−1^)	465.0	257.2	4.6	2234.4
SDI_max_ (# ha^−1^)	1058.9	436.1	110.6	6628.3
RD	0.42	0.15	0.01	1.00
**Time 2 (2013–2020; N = 94,401)**				
TPH (# ha^−1^)	1452.0	1376.1	2.5	72,625.2
QMD (cm)	18.1	9.8	2.5	166.4
BAPH (m^2^ ha^−1^)	23.9	14.3	0.1	275.8
pHW.BA	68.6	34.6	0.0	100.0
SDI (# ha^−1^)	481.5	255.8	4.6	2436.1
SDI_max_ (# ha^−1^)	1043.9	430.7	0.9	4464.6
RD	0.46	0.18	0.01	1.00

^a^TPH is trees per ha (stem density), QMD is quadratic mean diameter, BAPH is total basal area per ha, pHW.BA is the percentage of basal area in hardwood species, SDI is additive stand density index, SDI_max_ is the maximum stand density index, and RD is the relative density.

**Figure 3:**
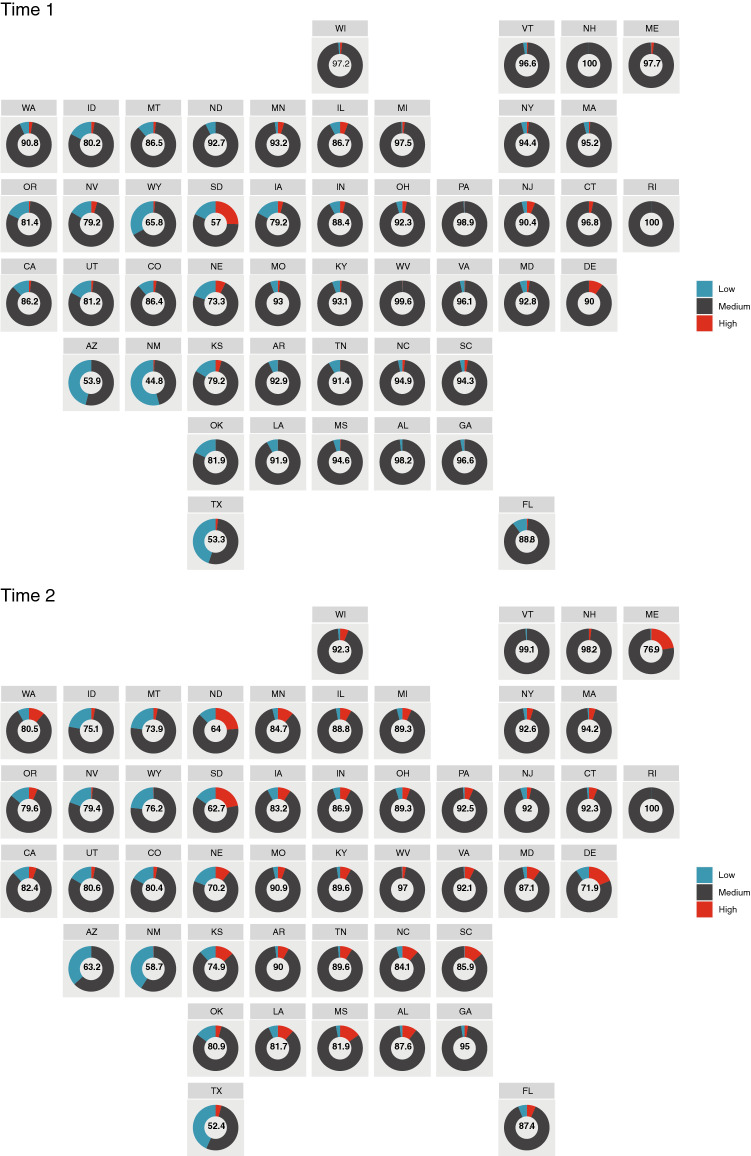
Proportion of forest area by stocking class and state for Time 1 (1999–2012) and Time 2 (2013–2020), with percentage of total forest area by state in fully stocked conditions (0.3 ≥ RD < 0.6) provided in the middle of each state doughnut. Facetted map produced in R v3.6.3^52^.

The apparent shift in US forest stand density to higher RD values is most evident in certain ecoregions, forest groups, and US states (see Supplemental Figures 4–8). For forest groups, the largest shifts were for aspen-birch, loblolly/shortleaf pine, oak-pine, redwood, and spruce-fir. For ecoregions, the largest shifts were for the Northern Lakes and Forests, Piedmont, Southeastern Plains, Southwestern Appalachians, and Willamette Valley. Almost all regions exhibited a slight shift towards a greater proportion of stands fully occupying their sites (e.g., Piedmont or Acadian Plains and Hills), while a minority of regions exhibited a slight shift towards low RD (e.g., Idaho Batholith, Middle Rockies, Chihuahuan Desert). States with the largest increase in high RD conditions were Alabama, Maine, Minnesota, Mississippi, and Wisconsin (Fig. 3). In contrast, Nebraska, South Dakota, Utah, and Wyoming had the largest increase in low RD conditions. These changes in levels of RD tended to propagate through the RD classes when substantial changes in RD were identified. For example, Arizona, New Mexico, and North Dakota all had the largest decreases in the proportion of their low RD forests, while they also had some of the largest increases in medium RD stands, which is a logical step in the forest stand development process. The same dynamic was witnessed in Maine, Louisiana, Mississippi, Alabama, and Washington: all had the largest reductions in the amount of forest land in the medium RD condition, but also had the biggest gains in their proportion of high RD forests.

**Figure 4:**
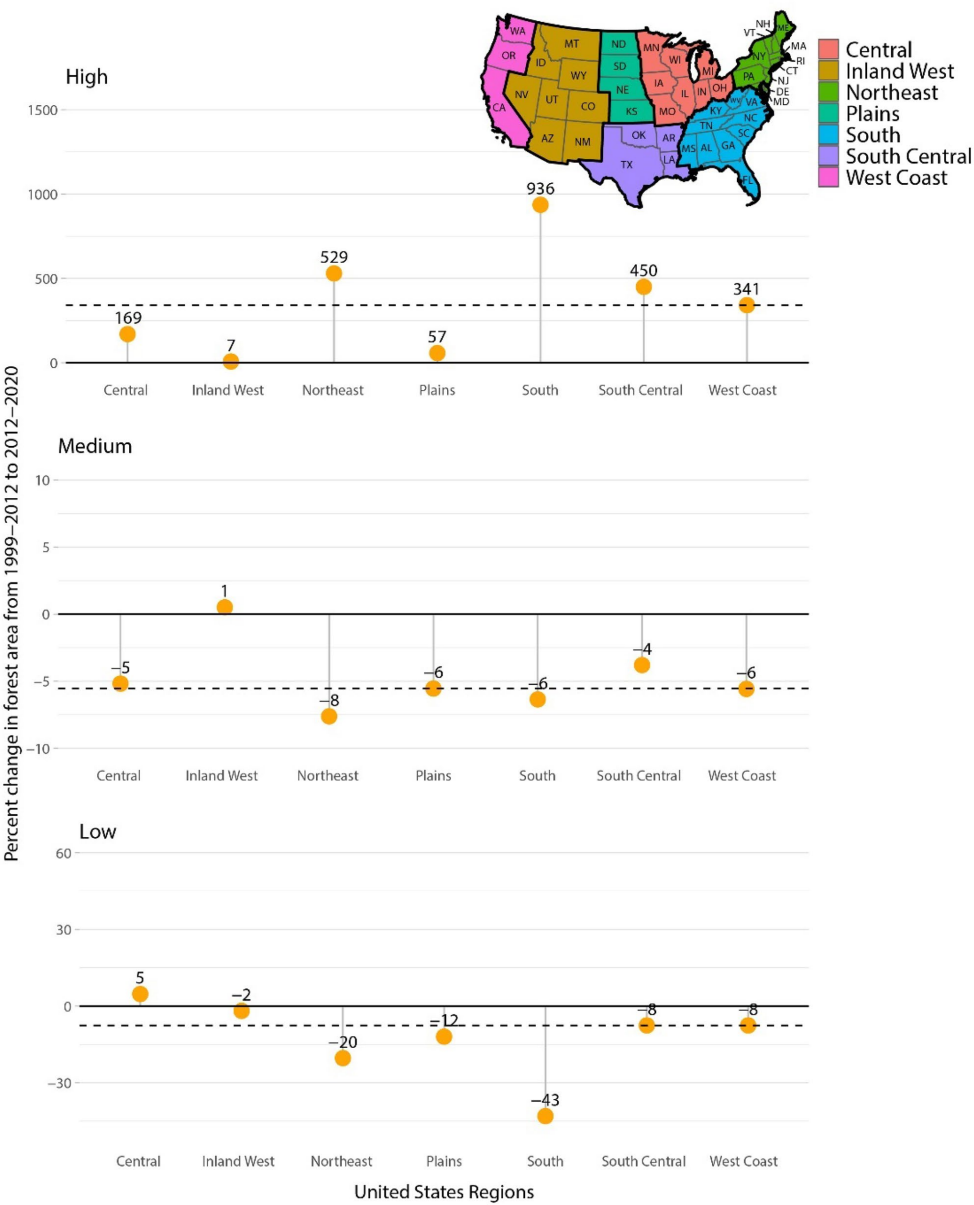
Percentage change in forest land area in low (0.00–0.30), medium (0.31–0.60), and high (0.61 +) relative density conditions by US region from Time 1 (1999–2012) to Time 2 (2013–2020). Dotted line denotes the median percentage change across all regions. Locator map produced in R v3.6.3^52^.

Changes in forest land area by RD classes and region perhaps provide a clearer picture of shifts in US forest size-density attributes (Fig. 4). The South has had some of the largest declines in the acreage of low RD forests (-43%), with concomitant large increases in high RD forest land area (936%). This stands in contrast to the Inland West of the US which had the lowest increase in high RD forest area (7%) among all US regions, which is coincident with widespread tree mortality^29^ and concurrent wildfires^30^ in parts of this region. Another interesting result is that almost all regions exhibited a wider dispersion of RD estimates at Time 2 compared to Time 1. This would be expected in the course of uninterrupted stand development because they contain a greater diversity (larger standard deviation of RD) of stand structures and/or size/density values across the landscapes. A point of caution regarding interpretation of forest RD results in ecosystems dominated by non-forest ecosystems is that the sampling error can be high relative to regions dominated by forests, but observed variability as assessed by the RD’s coefficient of variation was relatively consistent between Time 1 and 2 (Supplemental Figure 3).

**Figure 5:**
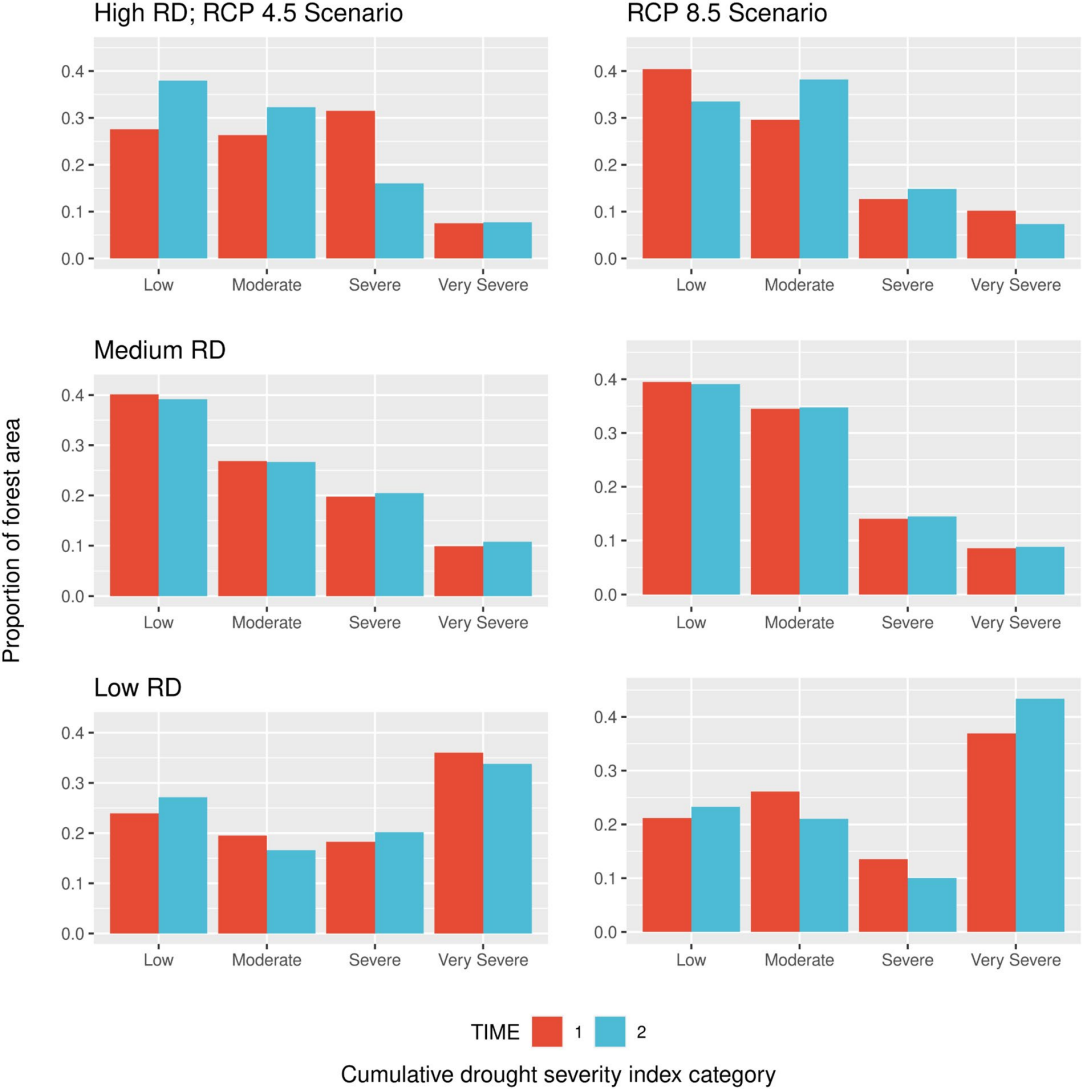
Proportion of coterminous US forest area by classes of live tree stocking (RD < 0.3 = low, 0.3 > RD < 0.6 = medium, RD > 0.6 = high) by classes of the projected cumulative drought severity index^31^ for 2040–2069 based on two regional climate projections (RCP 4.5 and 8.5). Drought categories were based on quartiles of the observed data (< 25% = low; > 25% & < 50% = moderate; > 50% & < 75% = severe; > 75% = very severe).

Classification of inventory plot observations by projected future cumulative drought severity indices^31^ indicated a rather stable distribution of high RD forest stands at both time periods by classes of projected future drought using the intermediate RCP 4.5 and high emission RCP 8.5 scenarios (Fig. 5). The most notable result was for low RD forests which had a plurality (~ 34% at time 2) of their observations in the most severe (Fig. 5, “very severe”) cumulative drought severity class for the RCP 4.5 scenario. For the RCP 8.5 scenario, this plurality of low RD acreage in the very severe drought index increased substantially to ~ 43%.

## Discussion

Assessing the status and trends of a forest size-density metric relative to a potential maximum (i.e., RD) is an additional tool for evaluation of forest establishment/management opportunities across large scales. Coincident with the contemporary maximum in forest land area potentially reached in the US^32^, US forests appear to be increasingly occupying their sites in terms of size-density relationships across most of the nation. Land use-change dynamics over the past 100 years, such as agricultural abandonment, has led to a substantial increase in forest land area and net carbon sequestration across much of the US^33^, with those stands established decades ago now reaching or exceeding medium levels of RD as highlighted in this analysis. Although this trend of increasing forest land area appears to have come to an end in the US^32^, the resulting forest stands stemming from decades of reforestation and/or lack of active timber management on reserved public lands or fragmented/urbanized forests has most likely resulted in forests that have a medium RD classification, where populations of live trees are approaching biologically relevant thresholds such as canopy closure and subsequent self-thinning-induced mortality. While the RD metric provides an objective quantification of the size-density status of tree populations comprising forest stands, various other metrics (e.g., remotely-sensed canopy change detection^34^ or landscape succession models^35^) and associated decision support tools are needed to appropriately frame RD in the context of the challenges^36^ facing contemporary forest assessment and management.

Forest metrics solely predicated on biological size-density metrics do not fully address degradation^37^ or carbon sequestration potentials^38^, but they do indicate the status of stand development and level of self-thinning in contemporary US forests should current management trends continue into the immediate future. Based on our analysis, 90% of US forests are now classified as having medium to high RD levels. In particular, the amount of stands with high RD levels has increased from ~ 1% of the total coterminous US forest land area to nearly 6% over the past 20 years, while stands with low levels of RD have slightly decreased. It should be noted that the stands with the lowest levels of RD (RD < 0.15) have slightly increased in areas of the west, which may be indicative of region-wide tree mortality and associated wildfires. Although medium-to-high RD stands might be more susceptible to disturbance events and/or tree mortality^27^, these stands continue to provide ecosystem services such as carbon sequestration, aesthetics/recreation, and/or habitat for biodiversity. Against a backdrop of static and/or declining forest land area and tree abundance coupled with increasing forest biomass and sawtimber volume (i.e., growth increments on larger trees), it is a logical extension to hypothesize that US lands classified as forests are currently sufficiently occupied with trees such that self-thinning and associated competition will be an increasingly important driver of future US forest dynamics. Although self-thinning is a normal progression of forest stand development, when viewed across broad landscapes it might indicate a lack of diversity in terms of stand age, species composition (i.e., lack of early seral species), and structure that can reduce the resilience of the forested landscape to future disturbances and potentially exclude a host of flora and fauna that rely primarily on early successional habitat^39^.

Forest management interventions to increase the resilience of these ecosystems to expected global change-induced disturbances (e.g., wildfires, droughts, and/or insects/disease) may need to explicitly address live tree density reduction across strategic-scales^40^. Without large-scale disturbances, improved low grade fiber markets, and/or forest management policy initiatives, we can expect the current trends of increasing RD to carry on as more stands continue their progression through stand development into high RD conditions (i.e., self-thinning–induced mortality) unless disturbances occur. At the same time, higher RD affords opportunities to increase the prevalence of ecological legacies (e.g., standing dead trees and/or old growth habitat) or more structurally complex forests across landscapes that may be essential under paradigms of adaptive management for the creation of resilient forests.

As evidenced by recent wide-scale forest disturbance events in the western US, a combination of long-term drought and high RD stands and/or stands succumbing to additional tree mortality events (e.g., insects/disease)^41^, the amount of forest in these intersecting sub-populations (i.e., projected future drought and current stand RD) has potentially been reduced with important implications for specific species^42^. At the same time, it is projected that high RD stands at Time 2 will be subject to more moderate or severe droughts. A somewhat differing result was found for low RD forests at Time 2, where greater than 40% were rated as “very severe” in RCP 8.5 future drought classifications. In a manner similar to stand development processes at the stand-level (i.e., developmental pathways advancing newly initiated forests through to medium RD levels), it can be hypothesized that coterminous US forests are collectively advancing along a developmental pathway with contemporary high RD stands in drought-prone areas already undergoing self-thinning and drought-induced mortality (e.g., wildfires) with the potential for substantial areas of forest to follow suit in the decades ahead. Because a large proportion of low RD stands are forecasted to be in future severe drought areas, their current size-density status may be more related to biological limits to stocking rather than strictly associated with management/policy premises. As a policy-focused framework, the relatively normal distribution of US forest RD suggests concomitant opportunities for reforestation (i.e., tree establishment) and forest management (i.e., density control) at opposing ends of the current distribution where ecologically and socially appropriate.

In terms of objectively quantifying forest stand RD, forest type classification and metrics of tree diameter variation (standard deviation and coefficients of variation) were the primary predictors of SDI_max_ and RD (Supplementary Figure 2). The original configurations of SDI and RD in past investigations were primarily at the stand-scale^21,43^, while more recent applications at regional^19,23,44^ and national^28^ scales have highlighted important predictors of a forest’s size/density metrics based on species-specific functional traits (e.g., wood density and/or shade tolerance) and/or climate (e.g., precipitation). It can be hypothesized that this study’s findings of forest type and ecological regions indicating the potential SDI_max_ that might be attained by any particular tree species configuration is synonymous with findings of climate and tree functional traits being influential in regards to how many trees of a certain species and size can occupy any given site. Although this study’s approach to quantifying the RD of forests is optimized for implementation and interpretation at large scales, the need remains for similar assessments to be downscaled to operational stand-level scales where functional traits of individual species may be a stronger driver. In order to affect management actions from the policy scale (e.g., continental to county) to operational (e.g., individual forest parcel), RD estimation procedures should be robust, temporally consistent, and complimentary. In addition to spatial scale, we hypothesize that RD formulations will need to be flexible to evolving tree species combinations and site changes (i.e., tree species migration and changing climate). Furthermore, refined interpretation and establishment of RD thresholds requires additional research across a host of ecosystem processes relevant to contemporary resource issues (e.g., wildlife habitat, carbon sequestration, or old growth maintenance). Should RD thresholds be dynamic to align with an array of forest ecosystem attributes (e.g., wildlife habitat or carbon stocks) and/or management objectives (e.g., resilience to disturbance^45^ or sawtimber yield)?

Based on this study’s assessment of tree density, nearly 90% of US forests are occupied with live trees at the point of canopy closure and beyond (i.e., medium to high RD) which aligns with estimates of static forest area and less tree abundance but greatly increasing tree volume and biomass. Rates of increase in RD have been disproportionately witnessed in the high RD stands, which have quintupled in terms of their forest land acreage over the past 20 years. Although this study did not evaluate the potential for reforestation and/or afforestation, the recent RD trends for land identified as being a forest land use suggest that active management^46^ may be equally important for climate change mitigation/adaptation efforts. In particular, the critical information outlined in this study may serve as a baseline assessment of current US forest RDs that can assist managers and policy makers with determining the optimal course of actions when it comes to assessing future forest carbon trajectories, land use planning, potential risks to disturbance, and strategies to increase the resilience of US forests in the face of global change. Relevant to forest C monitoring and associated markets, RD assessment may provide an objective metric for delineating forests where emission risk via disturbance may be high (i.e., high RD with high tree mortality risk and concomitant wildfire hazards) and where sequestration opportunities may be high (i.e., low RD with low tree mortality risk) and how these forest populations intersect across the coterminous US to achieve resiliency and provisioning of ecosystem services. Overall, the continual monitoring of these forest attributes and refinement of associated management applications across time and space is vital to ensure the effectiveness of any large-scale forest management efforts and policies.

## Methods

### Data

As the study area was the continental US, forest inventory data spanning the coterminous US was taken entirely from the public database of the USDA Forest Service’s Forest Inventory and Analysis (FIA) program. The FIA program is Congressionally authorized and appropriated to consistently inventory forests of the US on an annual basis^47^. As the FIA’s annual inventory program was first initiated circa 2000, robust forest resource change analysis is often focused on examining trends since 2000. FIA defines forest land as having at least 10% canopy cover of live tree species or the potential to support such cover if recently cut/disturbed along with a spatial size requirement of ~ 0.4 ha and at least ~ 36.6 m in width^17^. The national forestry inventory design is based on a spatially balanced sample of one plot every ca. 24 km^2^^48^. Each inventory plot consists of four points arranged in a cluster with one point at the center and three points oriented from the central subplot at 0°, 120°, and 240°^17^. Each of these four points are referred to as subplots. The distance from the center of the central point to the center of the surrounding points is 36.58 m. These four points form the center of fixed-area subplots used to tally live and standing dead trees (7.32 m plot radius, 12.7 cm minimum tree diameter). Numerous tree (e.g., diameter at breast height, species, height, and tree form) and site (e.g., elevation, slope, and aspect) attributes are measured by field crew at each plot. Furthermore, unique conditions (e.g., ownership, forest type, or stand age) at each plot are identified and mapped. Nested within this national plot design are variations in fixed area plot sizes and associated minimum tree diameters to accommodate the diversity of US forest ecosystems, including larger subplots termed “macroplots” (17.95 m radius, 12.7 cm minimum tree diameter) and smaller “microplots” (2.07 m radius, 2.54 cm minimum tree diameter). This variation in fixed-area plot sizes and minimum tree diameters was incorporated into estimation of stand attributes (subplot/macroplots vs plot level) across the scales of observation relevant to study objectives.

For the purpose of developing a maximum SDI (SDI_max_) model and evaluating change across time, the annual FIA national inventory plot observations were compiled into two datasets according to the oldest and most contemporary annual inventory by state (Supplementary Table 1). As the FIA inventory is implemented at the state-level (e.g., sample intensity and cycle length), the time period of the earliest annual inventory varies by state, but ranges between 1998 and 2013 for Time 1. For Time 2, the plot observations were measured between 2004 and 2020. For the sake of brevity across the majority of states that had a multi-year separation between annual inventories from the 2000s to the 2010s, the remeasurement time period is hereto referred to as “1999–2012” for Time 1 and “2013–2020” for Time 2. As the US annual forest inventory database is dynamically updated, the methods developed in this study may be applied to future inventories to actively monitor RD changes across a larger expanse of time and thus enabling more robust examination of forest change. Taken together, each collection of plots at each time period is considered an independent, systematically sampled observation of coterminous US forest conditions for that time period. As states may be sampled with varying sample intensities over varying remeasurement periods, average inventory year by state for each time period was used to standardize rates of decadal change. In order to estimate ecosystem-level changes in basic forest metrics of forest land area and live tree attributes (abundance, net saw log volume, and aboveground biomass), FIA’s population estimation procedures were used to expand each plot to a population estimate by ecosystem sections^49^ based on FIA’s post-stratification procedures. For this particular analysis, values were summarized at the FIA subplot-level to maintain observed variability and ensure more robust estimation of the plot-specific SDI_max_ value described further below.

### Statistical analysis

#### Determination of SDI_max_

A variety of approaches have been used to develop SDI_max_ models^50^. The primary challenges of SDI_max_ modeling are the need to determine a maximum rather than mean value, adequately addressing the often hierarchical or repeated nature of the data, and robustly assessing regional- or plot-specific relationships. The linear quantile mixed model (lqmm) approach first introduced by Andrews et al.^19^ produced the most biologically consistent, robust, and logical predictions of SDI_max_ while leveraging all available data when compared to other commonly used approaches^50^. For this reason and the nature of the available data described above, the lqmm approach^19^ was used here and is formulated as the Reineke^12^ size-density relationship:1where TPH is the stem density (# ha^−1^), QMD is the quadratic mean diameter (cm), b_i_ are fixed-effect parameters determined by lqmm, Ɛ_i_ is the residual for the ith plot, and ʋ_i_ and ɣ_i_ are the random-effect values for each ith plot also determined by lqmm. For this analysis, the 95th percentile was selected for determining both the fixed- and random-effects similar to Andrews et al.^18^ using the lqmm package^51^ in R v3.6.3^52^. To robustly determine a plot-level SDI_max_ value, Eq. (1) was fit using all available FIA subplot data, which would include between 4 and 8 observations per plot. Based on the implied maximum size-density relationship of Eq. (1) and a common index tree diameter (Reineke^12^ used 10 inches or 25.4 cm), a plot-specific SDI_max_ value is determined as:2

Consequently, a unique and plot-specific SDI_max_ value was determined from Eq. (2) and can then be combined with a plot-level estimate of SDI, which allows for the computation of RD (described further below) with direct implications for forest management. Based on the plot-specific SDI_max_ value, a variety of stand-, site-, and climate-related variables were determined based on prior findings^11,19,25^. For the stand-level attributes, this included measures of the diameter distribution (e.g., skewness, standard deviation, coefficient of variation, ratio of median to mean), species composition (e.g., ratio of hardwoods to softwoods, evenness), and functional traits (e.g., mean specific gravity), while site-level factors were primarily geographic and topographic factors like latitude/longitude, elevation, slope, and aspect. Climate was primarily based on 27 bioclimatic variables obtained from downscaled, 4 km resolution rasters from ClimateNA^53^. In addition to several continuous variables, numerous categorical variables were assessed, including FIA categories of forest group/type, site quality, ownership, reserve status, and distance from improved road as well as US EPA Level I-IV ecoregions^54^.

Given the number of variables available and the relatively high correlations among some of them, generalized boosted regression trees, a non-parametric statistical technique, was used to determine the relative influence of the factors^55^. A multi-level mixed-effects model accounting for spatial autocorrelations and potential residual heteroskedasticity was used to determine statistical significance of the most influential factors.

#### Determination of relative density (RD)

Using the predicted plot-level SDI_max_ value, RD was determined as the ratio between the tree-level additive estimate of stand density index^56^ and SDI_max_. Similar to SDI_max_, generalized boosted regression trees were used to determine the relative influence of various stand-, site-, and climate-related factors^55^. A multi-level mixed-effects model accounting for spatial autocorrelations and potential residual heteroskedasticity was used to determine statistical significance of the most influential factors. Based on expected stand dynamics, several critical RD zones are generally acknowledged and accepted^13,56^: (1) understocked related to full canopy closure (RD < 0.3); (2) full site occupancy with complete canopy closure (0.3 ≥ RD < 0.6); and (3) overstocked related to self-thinning induced mortality (RD ≥ 0.6).

#### Relative density summaries

Similar to Woodall et al.^28^, spatial trends in RD were examined by summarizing plot-level values to study hexagons (277.3 km^2^) across the US by Time 1 (1999–2012) and Time 2 (2013–2020). For both time periods, forest area population estimators^17^ were applied to plot-level estimates of RD to accommodate the varying forest inventory plot sampling intensity across time. Furthermore, to be consistent with past analyses^28,38^, plots were classified as understocked, fully stocked, and overstocked based on the critical RD zones described above. For the purpose of visualization, non-forest areas were removed from study hexagons using a national non-forest mask based on the most recent classified National Land Cover Dataset^57^. Population estimates of RD were summarized by state, forest type, EPA Level III ecoregions, and 30-year Cumulative Drought Severity Index^31^ (CDSI). For the latter, classification of severity was based on observed quantiles (< 25% = low; > 25% & < 50% = moderate; > 50% & < 75% = severe; > 75% = very severe). These were computed for one time period (2040–2069) and two RCPs (4.5 and 8.5).

All analyses and graphs were produced using R v3.6.3^52^. Statistical significance was assumed at *p* < 0.05 for all assessments.

## Supplementary Information

Supplementary Information.

## References

[1] Bonan GB (2008). Forests and climate change: Forcings, feedbacks, and the climate benefits of forests. Science.

[2] Pugh TAM (2019). Role of forest regrowth in global carbon sink dynamics. Proc. Natl. Acad. Sci. U. S. A..

[3] Allen CD (2010). A global overview of drought and heat-induced tree mortality reveals emerging climate change risks for forests. For. Ecol. Manag..

[4] Williams CA, Gu H, MacLean R, Masek JG, Collatz GJ (2016). Disturbance and the carbon balance of US forests: A quantitative review of impacts from harvests, fires, insects, and droughts. Glob. Planet. Change.

[5] Kurz WA (2008). Mountain pine beetle and forest carbon feedback to climate change. Nature.

[6] Lovett GM (2016). Nonnative forest insects and pathogens in the United States: Impacts and policy options. Ecol. Appl..

[7] Xu L (2021). Changes in global terrestrial live biomass over the 21st century. Sci Adv.

[8] Nave LE (2018). Reforestation can sequester two petagrams of carbon in US topsoils in a century. Proc. Natl. Acad. Sci. U. S. A..

[9] Millar CI, Stephenson NL, Stephens SL (2007). Climate change and forests of the future: Managing in the face of uncertainty. Ecol. Appl..

[10] McCarthy JK, Dwyer JM, Mokany K (2019). A regional-scale assessment of using metabolic scaling theory to predict ecosystem properties. Proc. Biol. Sci..

[11] Woodall CW, Miles PD, Vissage JS (2005). Determining maximum stand density index in mixed species stands for strategic-scale stocking assessments. For. Ecol. Manag..

[12] Reineke LH (1933). Perfecting a stand-density index for even-aged forests. J. Agric. Res..

[13] Long JN (1985). A practical approach to density management. For. Chron..

[14] Domke, G. *et al.* Forests. In *Second State of the Carbon Cycle Report (SOCCR2): A Sustained Assessment Report* (eds Cavallaro, N., Shrestha, G., Birdsey, R., Mayes, M. A., Najjar, R. G., Reed, S. C., Romero-Lankao, P. & Zhu, Z.) 365–398 (US Global Change Research Program, 2018).

[15] Yoda K, Kira T, Ogawa H, Hozumi K (1963). Self-thinning in overcrowded pure stands under cultivated and natural conditions. J. Biol. Osaka City Univ..

[16] Drew TJ, Flewelling JW (1979). Stand density management: An alternative approach and its application to Douglas-fir plantations. For. Sci..

[17] Bechtold, W. A. & Patterson, P. L. *The Enhanced Forest Inventory and Analysis Program: National Sampling Design and Estimation Procedures*. SRS GTR-80. USDA Forest Service, Southern Research Station, Asheville, North Carolina, USA. (2005). 10.2737/SRS-GTR-80.

[18] McGill BJ, Enquist BJ, Weiher E, Westoby M (2006). Rebuilding community ecology from functional traits. Trends Ecol. Evol..

[19] Andrews C, Weiskittel A, D’Amato AW, Simons-Legaard E (2018). Variation in the maximum stand density index and its linkage to climate in mixed species forests of the North American Acadian Region. For. Ecol. Manag..

[20] Nagel LM (2017). Adaptive silviculture for climate change: A national experiment in manager–scientist partnerships to apply an adaptation framework. J. For..

[21] Pretzsch H, Biber P (2005). A re-evaluation of the Reineke’s rule and stand density index. For. Sci..

[22] Condés S (2017). Climate influences on the maximum size-density relationship in Scots pine (*Pinus sylvestris* L.) and European beech (*Fagus sylvatica* L.) stands. For. Ecol. Manag..

[23] Ducey MJ, Woodall CW, Bravo-Oviedo A (2017). Climate and species functional traits influence maximum live tree stocking in the Lake States, USA. For. Ecol. Manag..

[24] Zhao D, Bullock BP, Montes CR, Wang M (2020). Rethinking maximum stand basal area and maximum SDI from the aspect of stand dynamics. For. Ecol. Manag..

[25] Weiskittel AR, Kuehne C (2019). Evaluating and modeling variation in site-level maximum carrying capacity of mixed-species forest stands in the Acadian Region of northeastern North America. For. Chron..

[26] Pretzsch H, del Río M (2020). Density regulation of mixed and mono-specific forest stands as a continuum: A new concept based on species-specific coefficients for density equivalence and density modification. For. Int. J. For. Res..

[27] Senf C, Buras A, Zang CS, Rammig A, Seidl R (2020). Excess forest mortality is consistently linked to drought across Europe. Nat. Commun..

[28] Woodall CW, Perry CH, Miles PD (2006). The relative density of forests in the United States. For. Ecol. Manag..

[29] Venturas MD, Todd HN, Trugman AT, Anderegg WRL (2020). Understanding and predicting forest mortality in the western United States using long-term forest inventory data and modeled hydraulic damage. New Phytol..

[30] Higuera PE, Abatzoglou JT (2021). Record-setting climate enabled the extraordinary 2020 fire season in the western United States. Glob. Change Biol..

[31] Peters, M. P. & Iverson, L. R. Projected drought for the conterminous United States in the 21st century. In *Effects of Drought on Forests and Rangelands in the United States* (eds Vose, J. M., Peterson, D. L., Luce, C. H. & Patel-Weynand, T.) vol. Gen. Tech. Rep. WO-98 19–39 (USDA Forest Service, 2019).

[32] Coulston JW, Woodall CW, Domke GM, Walters BF (2016). Refined forest land use classification with implications for United States national carbon accounting. Land Use Policy.

[33] Wear DN, Coulston JW (2015). From sink to source: Regional variation in U.S. forest carbon futures. Sci. Rep..

[34] Senf C, Sebald J, Seidl R (2021). Increasing canopy mortality affects the future demographic structure of Europe’s forests. One Earth.

[35] Morin X, Fahse L, Scherer-Lorenzen M, Bugmann H (2011). Tree species richness promotes productivity in temperate forests through strong complementarity between species. Ecol. Lett..

[36] Griscom BW (2017). Natural climate solutions. Proc. Natl. Acad. Sci. U. S. A..

[37] Gunn JS, Ducey MJ, Belair E (2019). Evaluating degradation in a North American temperate forest. For. Ecol. Manag..

[38] Domke GM, Oswalt SN, Walters BF, Morin RS (2020). Tree planting has the potential to increase carbon sequestration capacity of forests in the United States. Proc. Natl. Acad. Sci. U. S. A..

[39] King DI, Schlossberg S (2014). Synthesis of the conservation value of the early-successional stage in forests of eastern North America. For. Ecol. Manag..

[40] Stephens SL (2020). Forest restoration and fuels reduction: Convergent or divergent?. Bioscience.

[41] Berner LT, Law BE, Meddens AJH, Hicke JA (2017). Tree mortality from fires, bark beetles, and timber harvest during a hot and dry decade in the western United States (2003–2012). Environ. Res. Lett..

[42] Stanke H, Finley AO, Domke GM, Weed AS, MacFarlane DW (2021). Over half of western United States’ most abundant tree species in decline. Nat. Commun..

[43] Weiskittel AR, Gould PJ, Temesgen H (2009). Sources of variation in the self-thinning boundary line for three species with varying levels of shade tolerance. For. Sci..

[44] Ducey MJ, Knapp RA (2010). A stand density index for complex mixed species forests in the northeastern United States. For. Ecol. Manag..

[45] Kurz WA, Stinson G, Rampley GJ, Dymond CC, Neilson ET (2008). Risk of natural disturbances makes future contribution of Canada’s forests to the global carbon cycle highly uncertain. Proc. Natl. Acad. Sci. U. S. A..

[46] Seidl R, Schelhaas M-J, Lexer MJ (2011). Unraveling the drivers of intensifying forest disturbance regimes in Europe. Glob. Change Biol..

[47] Nelson, M. D. *et al.* Defining the United States land base: A technical document supporting the USDA Forest Service 2020 RPA assessment. In *Gen. Tech. Rep. NRS-191*, Vol. 191, 1–70 (2020).

[48] Patterson, P. L. & Reams, G. A. Combining panels for forest inventory and analysis estimation. *Gen. Tech. Rep. SRS-80. Asheville, NC: US Department of Agriculture, Forest Service, 79–84* (2005).

[49] Bailey RG (1983). Delineation of ecosystem regions. Environ. Manag..

[50] Salas-Eljatib C, Weiskittel AR (2018). Evaluation of modeling strategies for assessing self-thinning behavior and carrying capacity. Ecol. Evol..

[51] Geraci M (2013). Linear quantile mixed models: The lqmm package for Laplace quantile regression. J. Stat. Softw..

[52] R Development Core Team (2019). R: A Language and Environment for Statistical Computing.

[53] Wang T, Hamann A, Spittlehouse D, Carroll C (2016). Locally downscaled and spatially customizable climate data for historical and future periods for North America. PLoS ONE.

[54] Omernik JM, Griffith GE (2014). Ecoregions of the conterminous United States: Evolution of a hierarchical spatial framework. Environ. Manag..

[55] De’ath G (2007). Boosted trees for ecological modeling and prediction. Ecology.

[56] Long JN, Daniel TW (1990). Assessment of growing stock in uneven-age stands. West. J. Appl. For..

[57] Yang L (2018). A new generation of the United States National Land Cover Database: Requirements, research priorities, design, and implementation strategies. ISPRS J. Photogramm. Remote Sens..

