# Supplementary material for: Relative density of United States forests has shifted to higher levels over last two decades with important implications for future dynamics
Source: Sci Rep. 2021 Sep 22;11:18848. doi: 10.1038/s41598-021-98244-w (PMC8458300; doi:10.1038/s41598-021-98244-w)
Supplement: Supplementary file 1 — Supplementary Information. [file 41598_2021_98244_MOESM1_ESM.pdf]

Supplementary Materials

Supplementary Table S1. Summary of forest inventories by state and inventories

State	Time 1		Time 2	
	FIADB Evaluation ID	Inventory Years	FIADB Evaluation ID	Inventory Years
Alabama	10401	2000-2004	12001	2013-2020
Arizona	41001	2001-2010	41901	2010-2019
Arkansas	50901	2000-2009	51901	2014-2019
California	61001	2002-2010	61901	2010-2019
Colorado	81001	2002-2010	81901	2010-2019
Connecticut	90701	2003-2007	91901	2013-2019
Delaware	100801	2004-2008	101901	2013-2019
Florida	121201	2002-2012	121701	2012-2017
Georgia	130801	1998-2008	131801	2014-2018
Idaho	161201	2004-2012	161801	2009-2018
Illinois	170501	2001-2005	171901	2012-2019
Indiana	180301	1999-2003	181901	2013-2019
Iowa	190301	1999-2003	191901	2013-2019

State	Time 1		Time 2	
	FIADB Evaluation ID	Inventory Years	FIADB Evaluation ID	Inventory Years
Kansas	200501	2001-2005	201901	2013-2019
Kentucky	210501	2000-2005	211701	2012-2017
Louisiana	221201	2001-2012	221701	2009-2017
Maine	230601	2002-2006	231901	2015-2019
Maryland	240801	2004-2008	241901	2013-2019
Massachusetts	250701	2003-2007	251901	2013-2019
Michigan	260401	2000-2004	261901	2013-2019
Minnesota	270301	1999-2003	271901	2015-2019
Mississippi	281201	2006-2012	281901	2012-2019
Missouri	290301	1999-2003	291901	2013-2019
Montana	301001	2003-2010	301901	2010-2019
Nebraska	310501	2001-2005	311901	2013-2019
Nevada	321201	2004-2012	321801	2009-2018
New Hampshire	330701	2002-2007	331901	2013-2019
New Jersey	340801	2004-2008	341901	2015-2019

State	Time 1		Time 2	
	FIADB Evaluation ID	Inventory Years	FIADB Evaluation ID	Inventory Years
New Mexico	351201	2005-2013	351801	2009-2018
New York	360701	2002-2007	361901	2013-2019
North Carolina	370601	2002-2006	371901	2011-2019
North Dakota	380501	2001-2005	381901	2013-2019
Ohio	390601	2001-2006	391901	2013-2019
Oklahoma	401201	2008-2012	401801	2009-2018
Oregon	411001	2001-2010	411901	2008-2019
Pennsylvania	420601	2002-2006	421901	2013-2019
Rhode Island	440701	2003-2007	441901	2013-2019
South Carolina	450501	1999-2005	451801	2014-2018
South Dakota	460501	2001-2005	461901	2013-2019
Tennessee	470301	1999-2003	471701	2012-2017
Texas	480701	2001-2007	481701	2004-2017
Utah	490901	2000-2009	491901	2010-2019
Vermont	500701	2003-2007	501901	2013-2019

State	Time 1		Time 2	
	FIADB Evaluation ID	Inventory Years	FIADB Evaluation ID	Inventory Years
Virginia	510601	1998-2006	511801	2014-2018
Washington	531101	2002-2011	531901	2008-2019
West Virginia	540801	2004-2008	541901	2013-2019
Wisconsin	550401	2000-2004	551901	2013-2019
Wyoming	560001	2000	561901	2011-2019

Supplementary Table 2. Summary attributes of maximum SDI (SDI_{max}) subplot-level observations (*N* = 1,257,773) used for developing the size-density relationship of Equation [1].

**Attribute**[1]	**Mean**	**SD**	**Minimum**	**Maximum**
TPH (# ha^{-1})	1,6812.3	2,041.7	2.1	64,352.3
QMD (cm)	19.8	12.2	2.54	284.7
BAPH (m^2 ha^{-1})	26.4	19.2	0.4	484.1
SDI (# ha^{-1})	539.5	348.1	0.9	3,220.6
SDI_{max} (# ha^{-1})	1,038.8	422.8	95.3	4,521.8
RD	0.49	0.14	0.04	1.0

[1]TPH is trees per hectare, QMD is quadratic mean diameter, BAPH is total basal area per ha, SDI is additive stand density index, SDI_{max} is the maximum stand density index, and RD is the relative density.

Supplementary Table 3. Area by relative density stocking level and time based on ecologically significant and management-oriented thresholds (e.g., self-thinning induced mortality) based on the values used in Woodall et al.[28].

**Relative density Class**	**Time 1 (1999-2012)**		**Time 2 (2013-2020)**	
	**Total forest area (million ha)**	**Percentage of total forest area**	**Total forest area (million ha)**	**Percentage of total forest area**
0.00–0.15	4.10	1.51	4.71	1.73
0.16–0.30	21.48	7.87	20.23	7.43
0.31–0.45	152.56	55.84	103.33	37.94
0.46–0.60	92.39	33.82	128.42	47.15
0.61–0.75	2.37	0.87	14.68	5.39
0.76+	0.29	0.11	0.99	0.36
Total	273.19	100.0	272.36	100.0

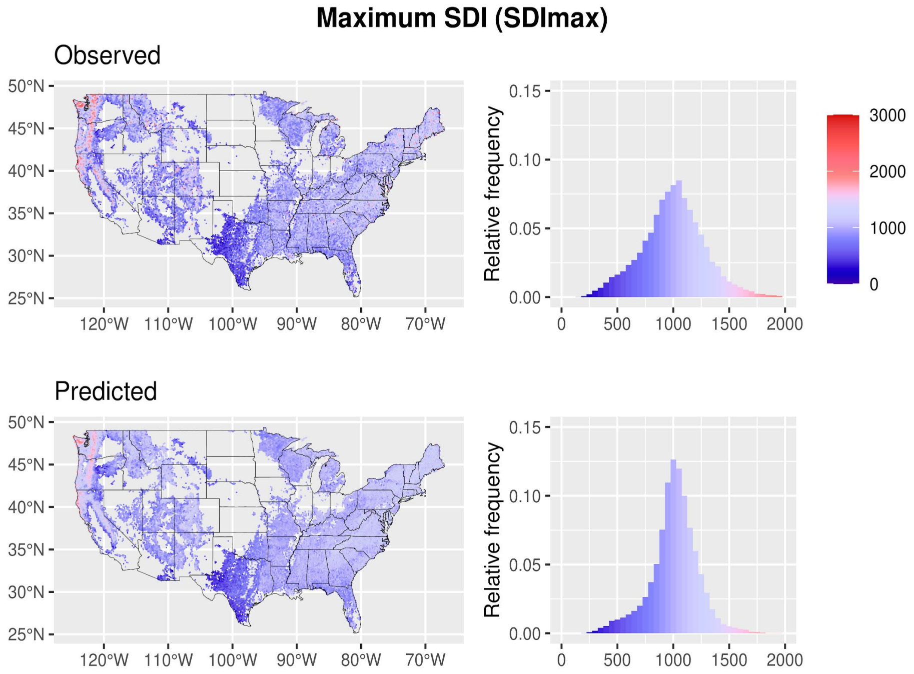

a)

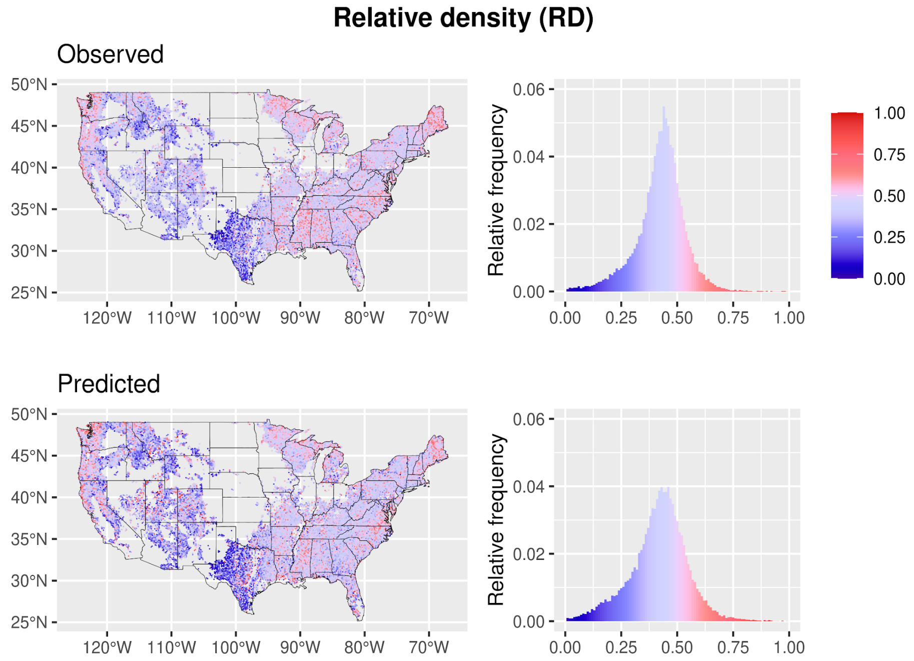

b)

Supplementary Figure 1. National map of (a) observed and predicted maximum SDI (SDI_{max}) and (b) relative density (RD) predicted and observed by hexagon (277.3 km^2). Map produced in R v3.6.3[52], R Development Core Team. R: A Language and Environment for Statistical Computing. URL: https://www.r-project.org/

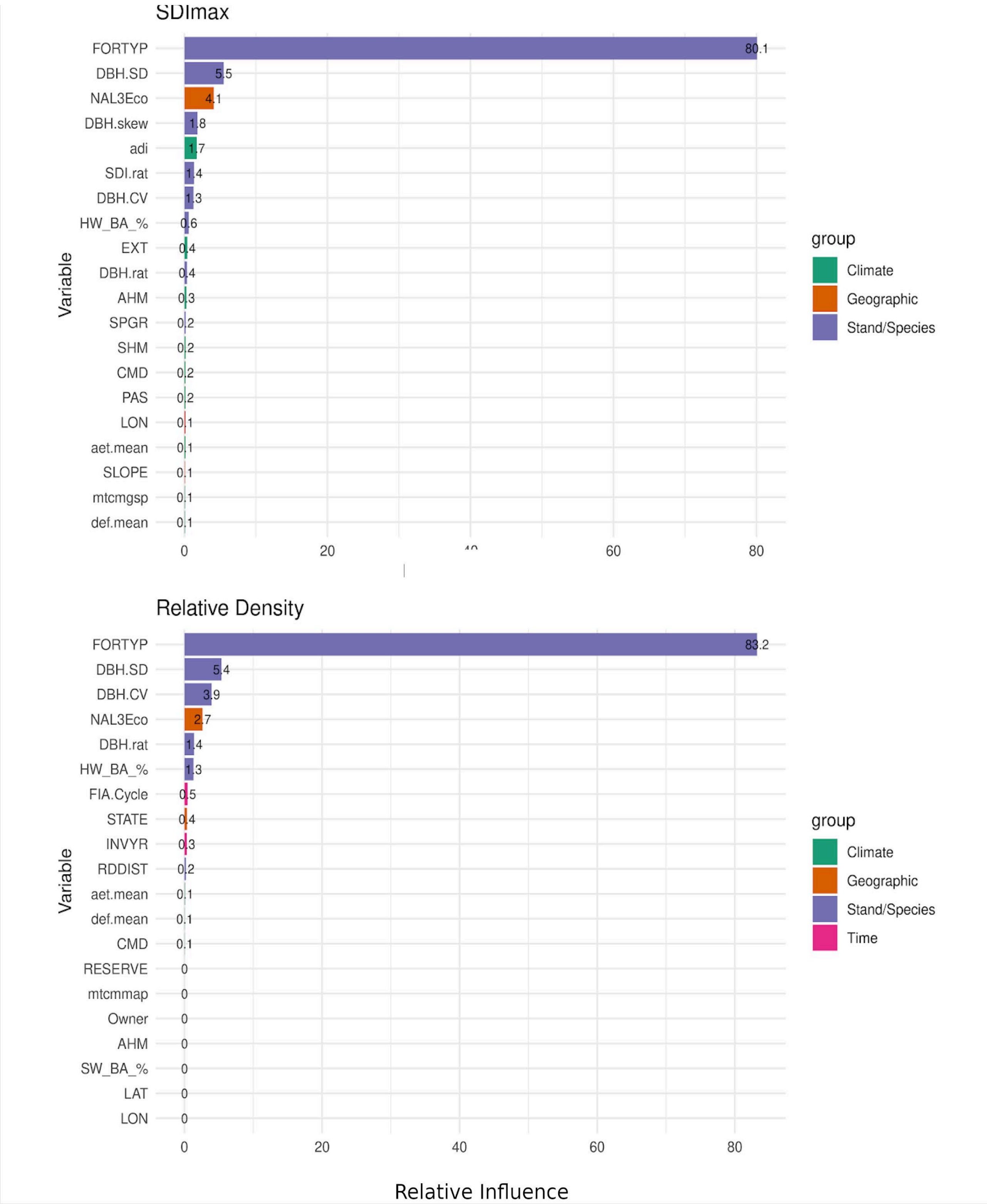

Supplementary Figure 2. Relative influence of various stand-, site-, topography- and climate-related factors on maximum SDI (SDI_{max}) and relative density (RD) as determined by generalized boosted regression trees. The key variables included FIA forest type (FORTYP), standard deviation of the tree

diameter (DBH; DBH.SD), the North American Level III Ecoregions (NAL3Eco), skewness of the DBH distribution (DBH.skew), annual dryness index (adi; Degree-days >5°C divided by mean annual precipitation), ratio of additive to traditional SDI (SDI.rat), coefficient variation of DBH (DBH.CV), hardwood species proportion of the basal area (HW_BA_%), softwood species proportion of the basal area (SW_BA_%), extreme maximum temperature (EXT) between 1961-1990, the ratio between the mean and median DBH (DBH.rat), the annual heat-moisture index (AHM; (mean annual temperature+10)/(mean annual precipitation/1000)), mean specific gravity (SPGR), summer heat-moisture index (SHM; (mean temperature of the warmest month)/(mean summer precipitation/1000)), Hargreaves climatic moisture deficit (CMD), precipitation as snow (PAS), longitude (LON), mean annual evapotranspiration (aet.mean), percent slope (SLOPE), interaction between the mean temperature of coldest month (mtcm) and growing season precipitation (gsp; mtcmgsp), climate water deficit mean between 2000-2009 (def.mean), latitude (LAT), the FIA cycle number (FIA.Cycle; Time 1 vs. Time 2), US state (STATE), the inventory year (INVYR), distance to a major road FIA classification (RDDIST), ecological research status (RESERVE), and FIA forestland ownership classification (Owner).

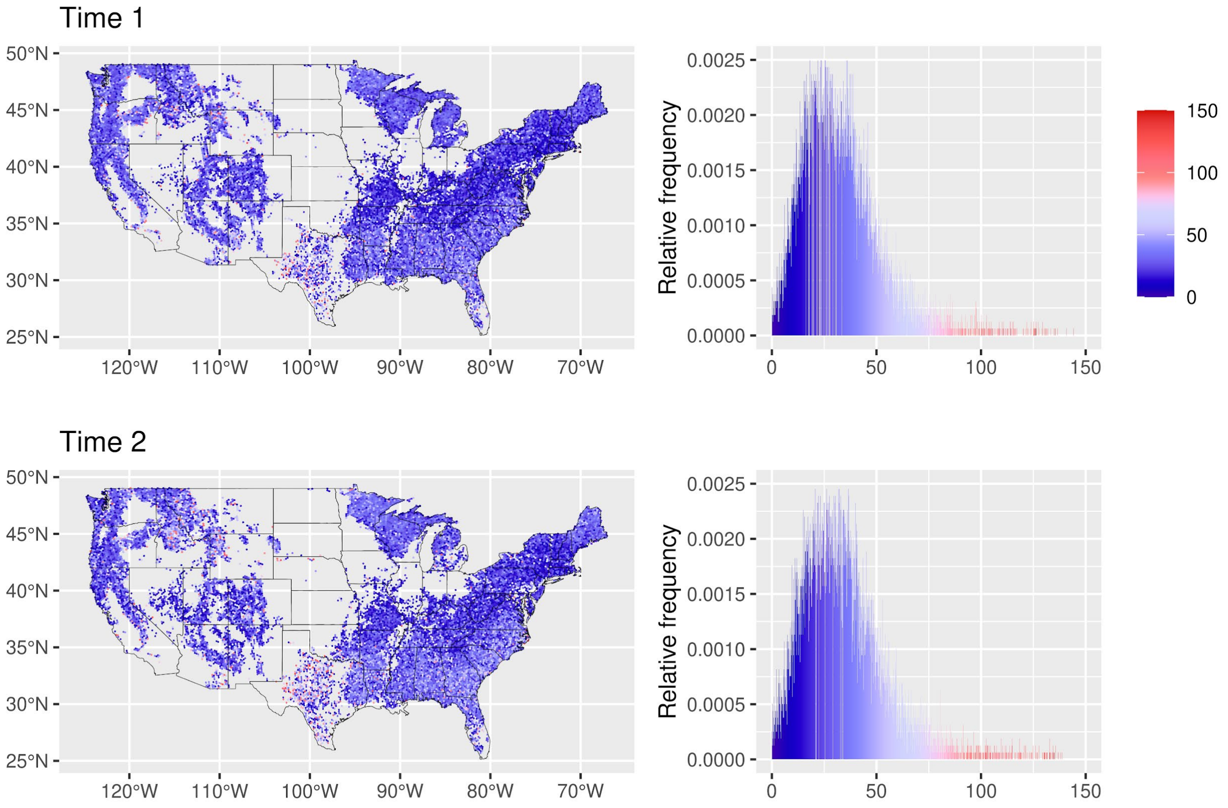

Supplementary Figure 3. Coefficient of variation (CV) of observed relative density (RD) by hexagon (277.3 km^2) at Times 1 and 2. Map produced in R v3.6.3[52], R Development Core Team. R: A Language and Environment for Statistical Computing. URL: https://www.r-project.org/

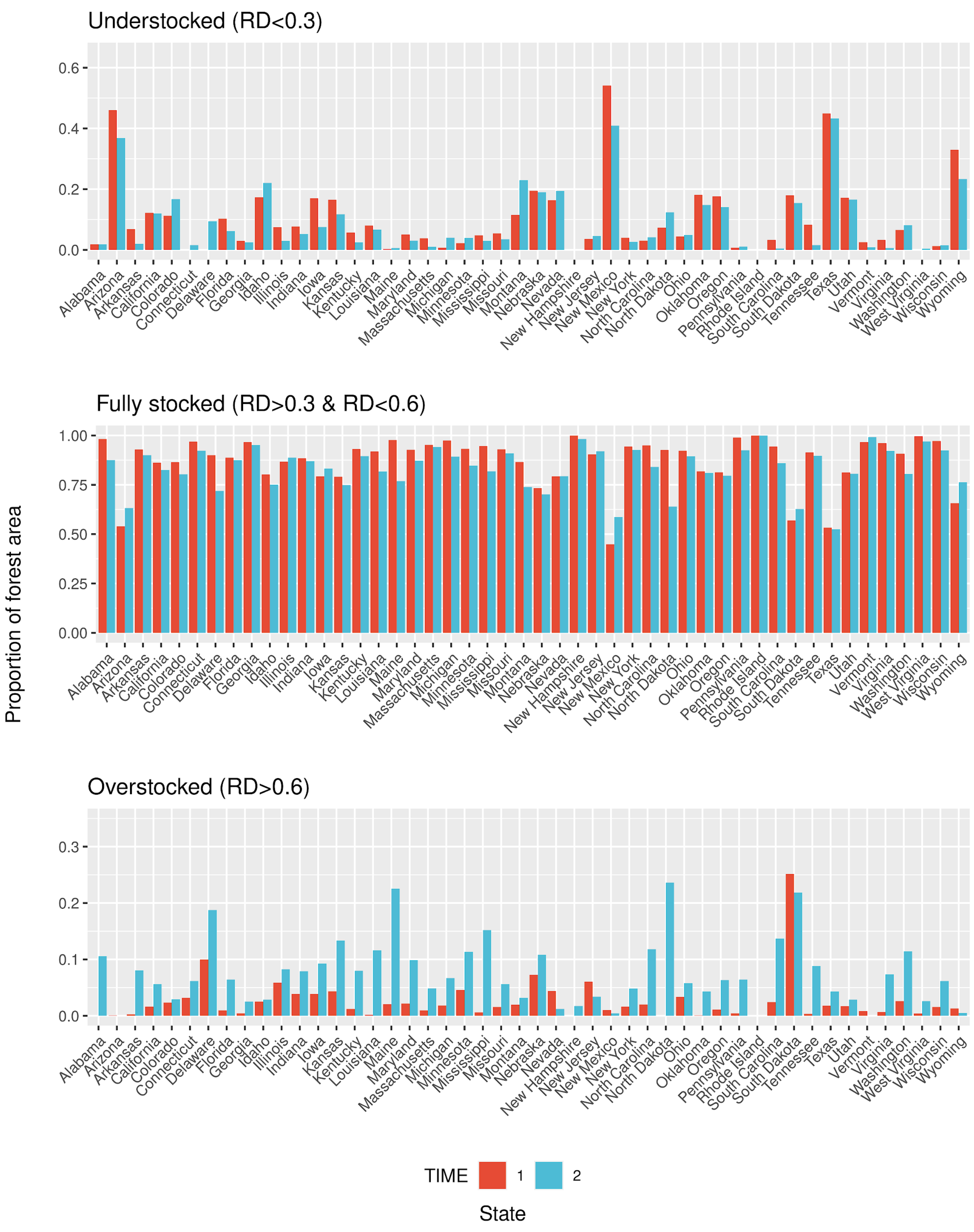

Supplementary Figure 4. Proportion of total forest area by classes of relative density (RD), time period, and state for coterminous US forests.

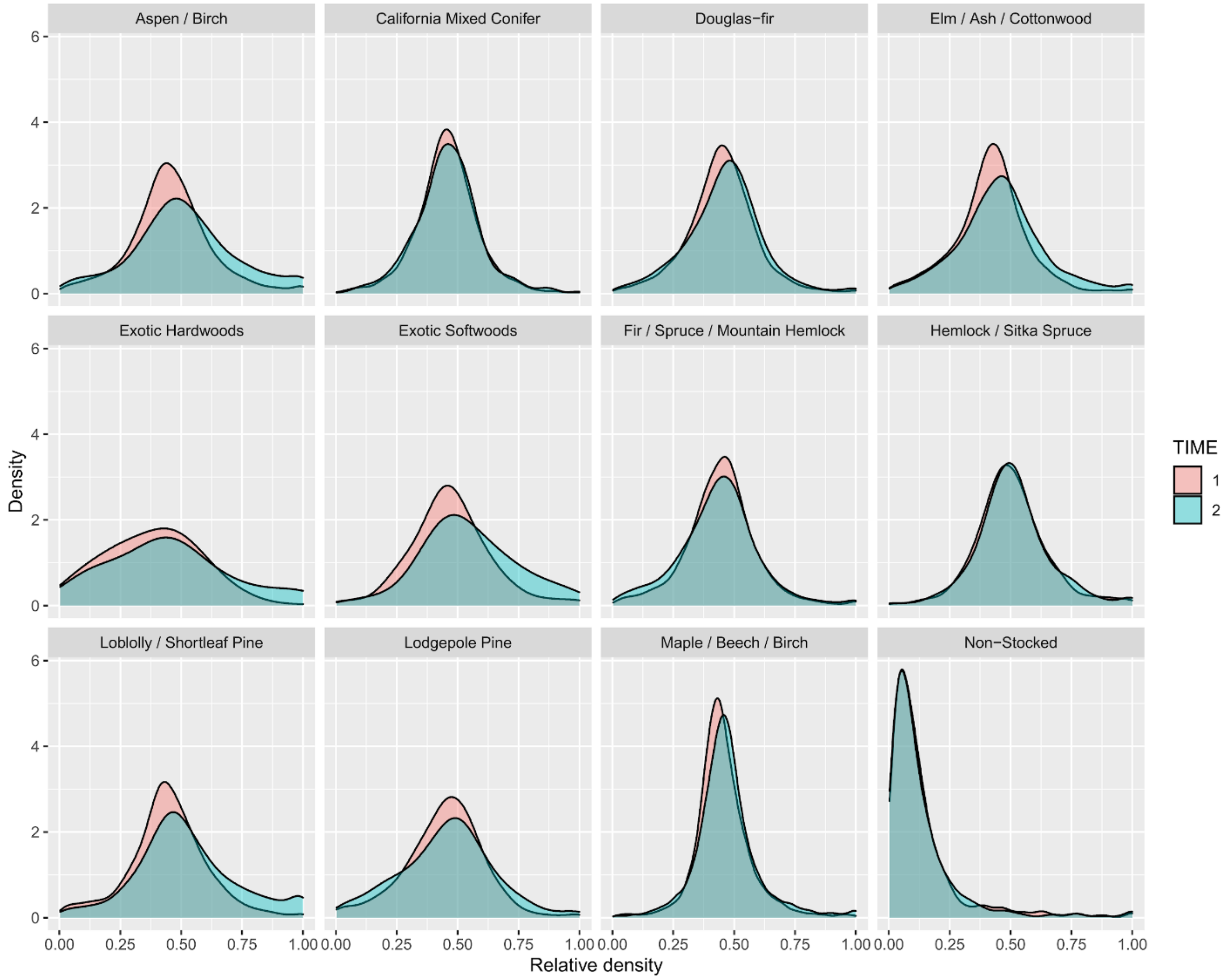

Aspen / Birch
California Mixed Conifer
Douglas−fir
Elm / Ash / Cottonwood
Exotic Hardwoods
Exotic Softwoods
Fir / Spruce / Mountain Hemlock
Hemlock / Sitka Spruce
Loblolly / Shortleaf Pine
Lodgepole Pine
Maple / Beech / Birch
Non−Stocked
Density
Relative density
0.00
0.25
0.50
0.75
1.00
0
2
4
6
TIME
1
2

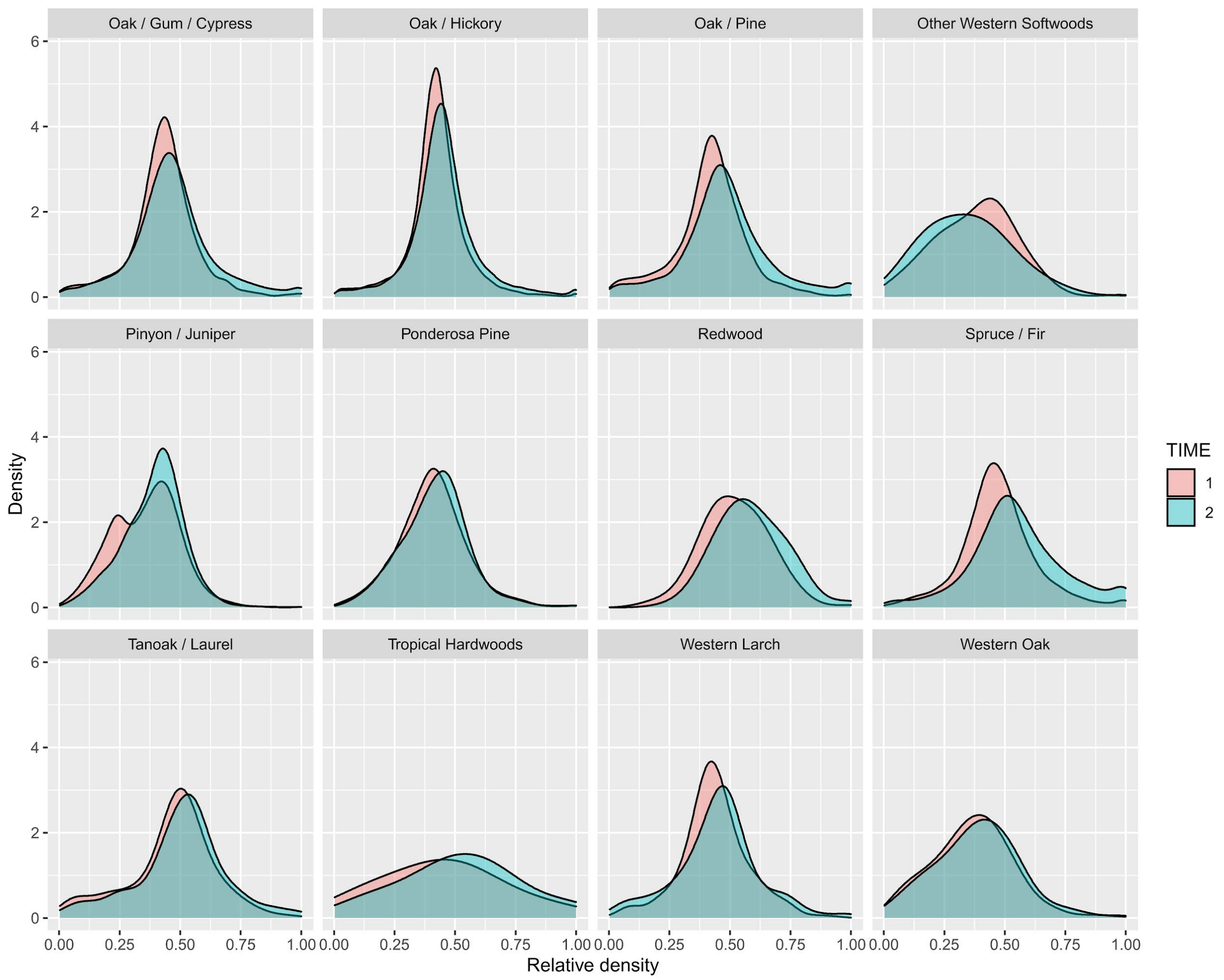

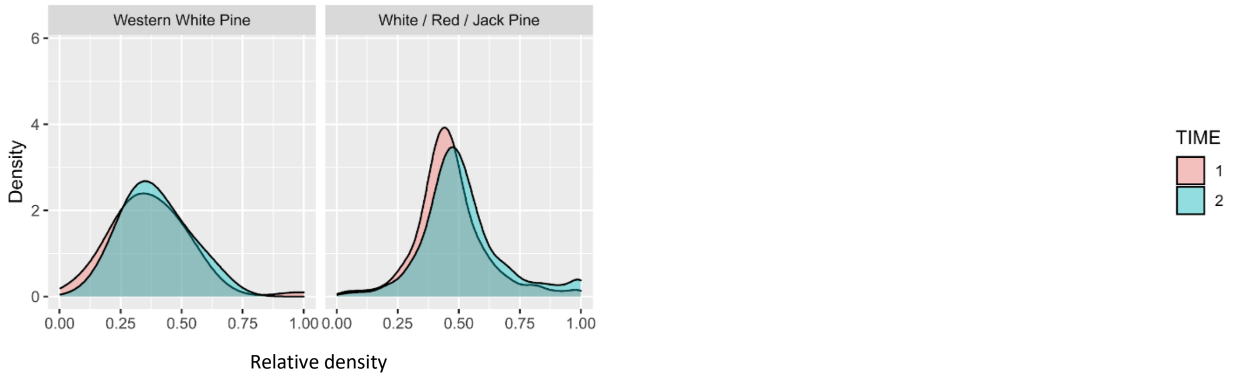

Supplementary Figure 5. Relative frequency distribution of relative density (RD) for Time 1 and 2 by US Forest Service, Forest Inventory and Analysis (FIA) forest group.

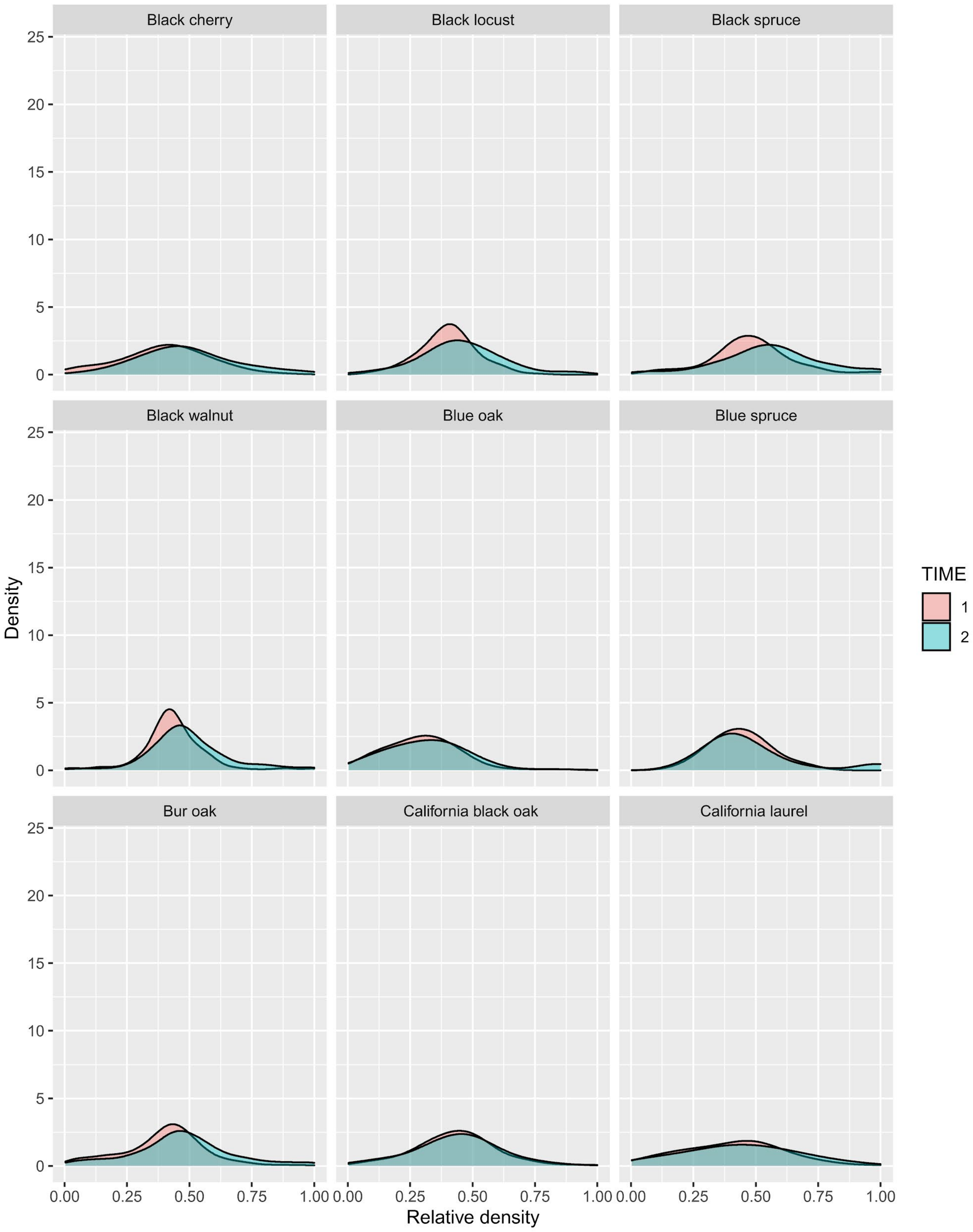

Black cherry
Black locust
Black spruce
Black walnut
Blue oak
Blue spruce
Bur oak
California black oak
California laurel
Density
Relative density
TIME
1
2
0
5
10
15
20
25
0.00
0.25
0.50
0.75
1.00

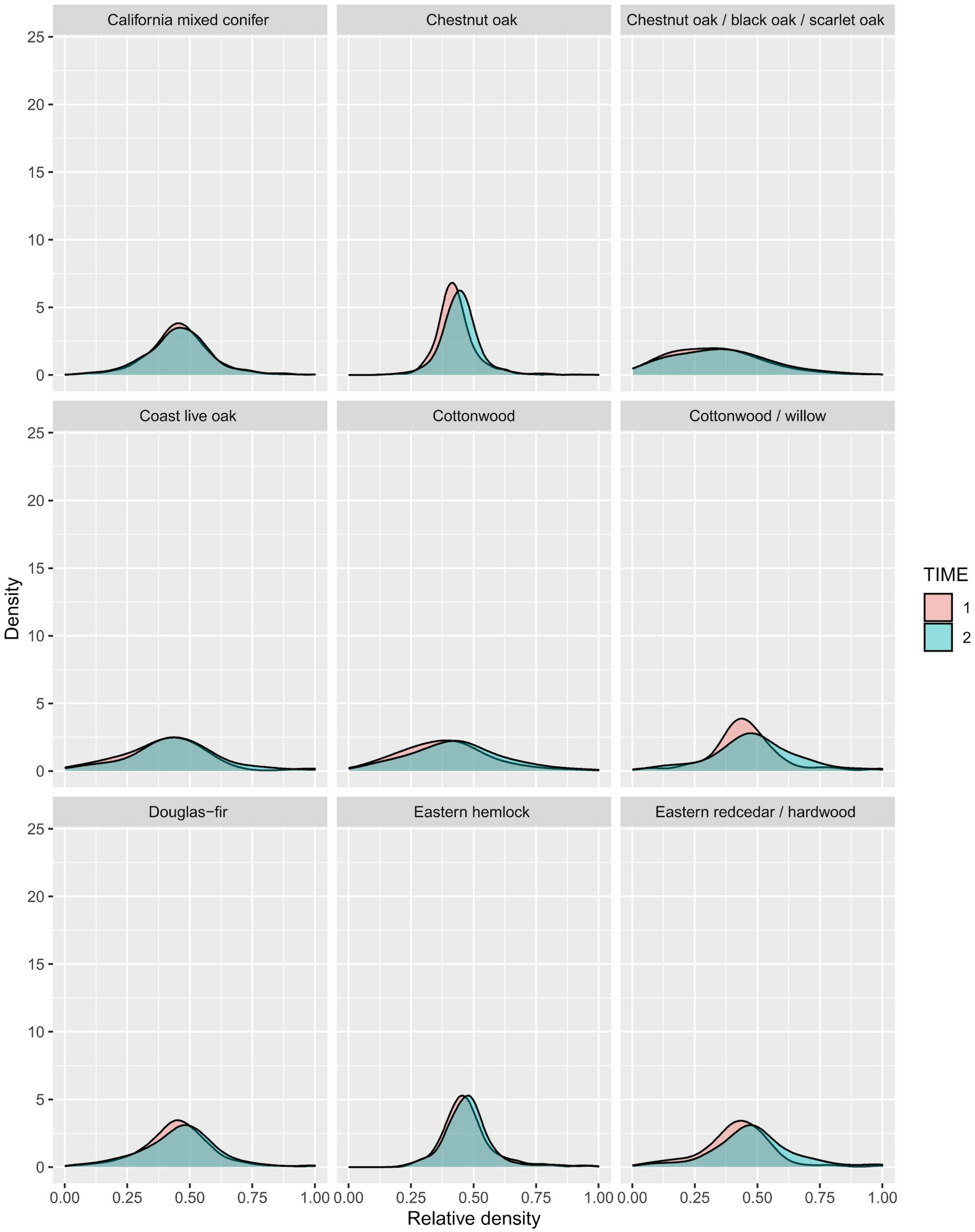
California mixed conifer
Chestnut oak
Chestnut oak / black oak / scarlet oak
Coast live oak
Cottonwood
Cottonwood / willow
Douglas−fir
Eastern hemlock
Eastern redcedar / hardwood
Density
Relative density
TIME
1
2
0
5
10
15
20
25
0.00
0.25
0.50
0.75
1.00

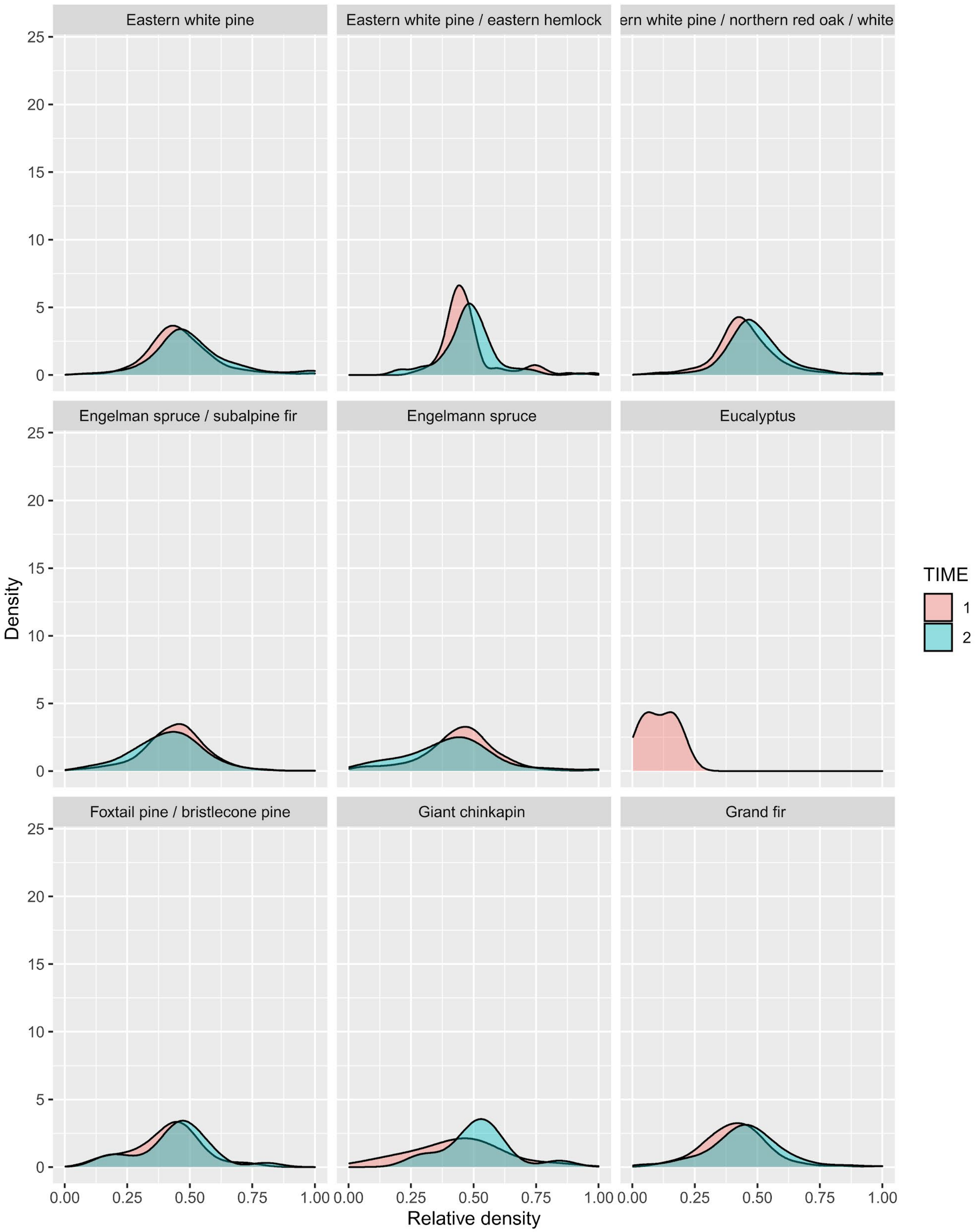

Eastern white pine
Eastern white pine / eastern hemlock
ern white pine / northern red oak / white
Engelman spruce / subalpine fir
Engelmann spruce
Eucalyptus
Foxtail pine / bristlecone pine
Giant chinkapin
Grand fir
Density
Relative density
TIME
1
2
0
5
10
15
20
25
0.00
0.25
0.50
0.75
1.00

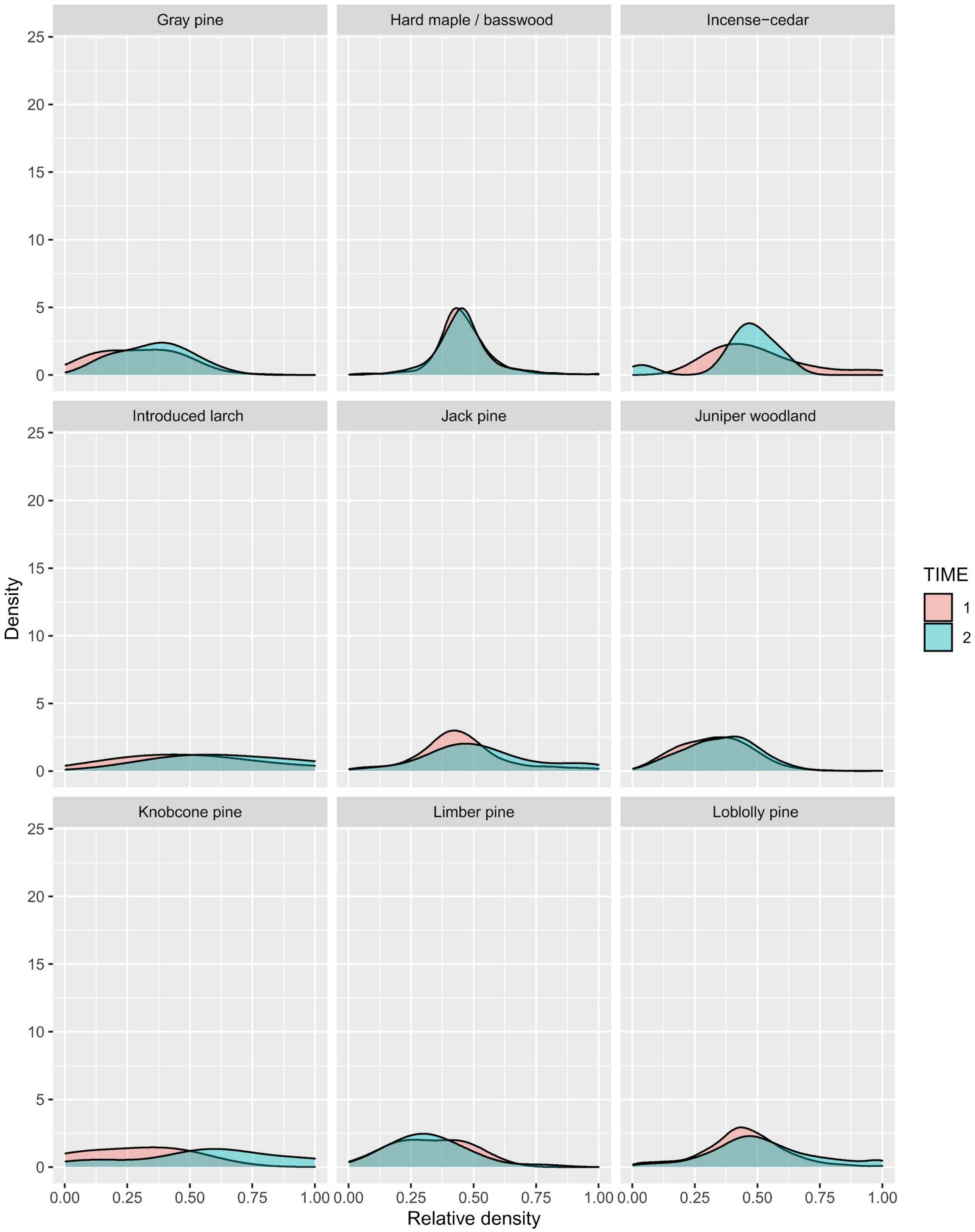
Gray pine
Hard maple / basswood
Incense−cedar
Introduced larch
Jack pine
Juniper woodland
Knobcone pine
Limber pine
Loblolly pine
Density
Relative density
TIME
1
2
0
5
10
15
20
25
0.00
0.25
0.50
0.75
1.00

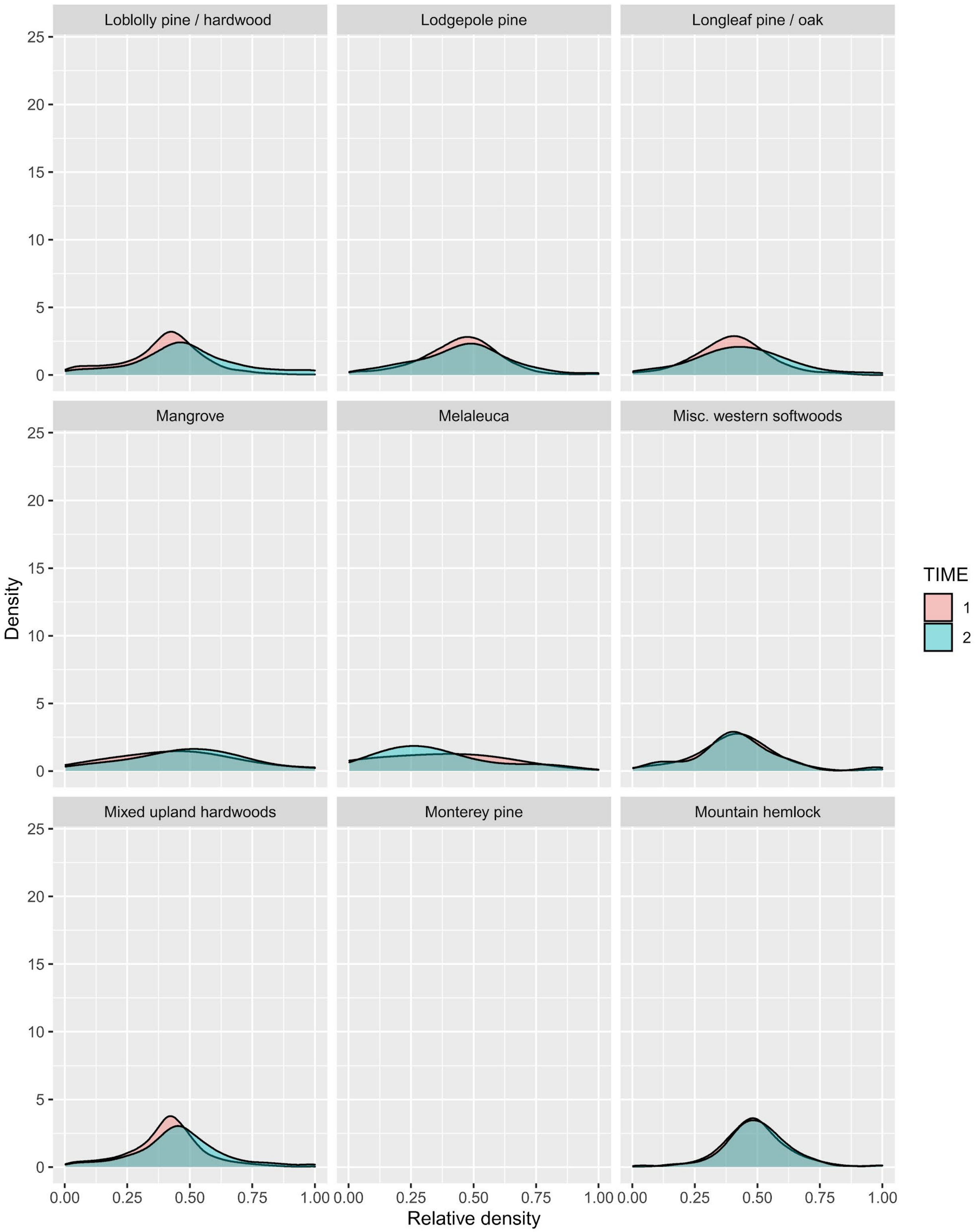

Loblolly pine / hardwood
Lodgepole pine
Longleaf pine / oak
Mangrove
Melaleuca
Misc. western softwoods
Mixed upland hardwoods
Monterey pine
Mountain hemlock
Density
Relative density
TIME
1
2
0
5
10
15
20
25
0.00
0.25
0.50
0.75
1.00

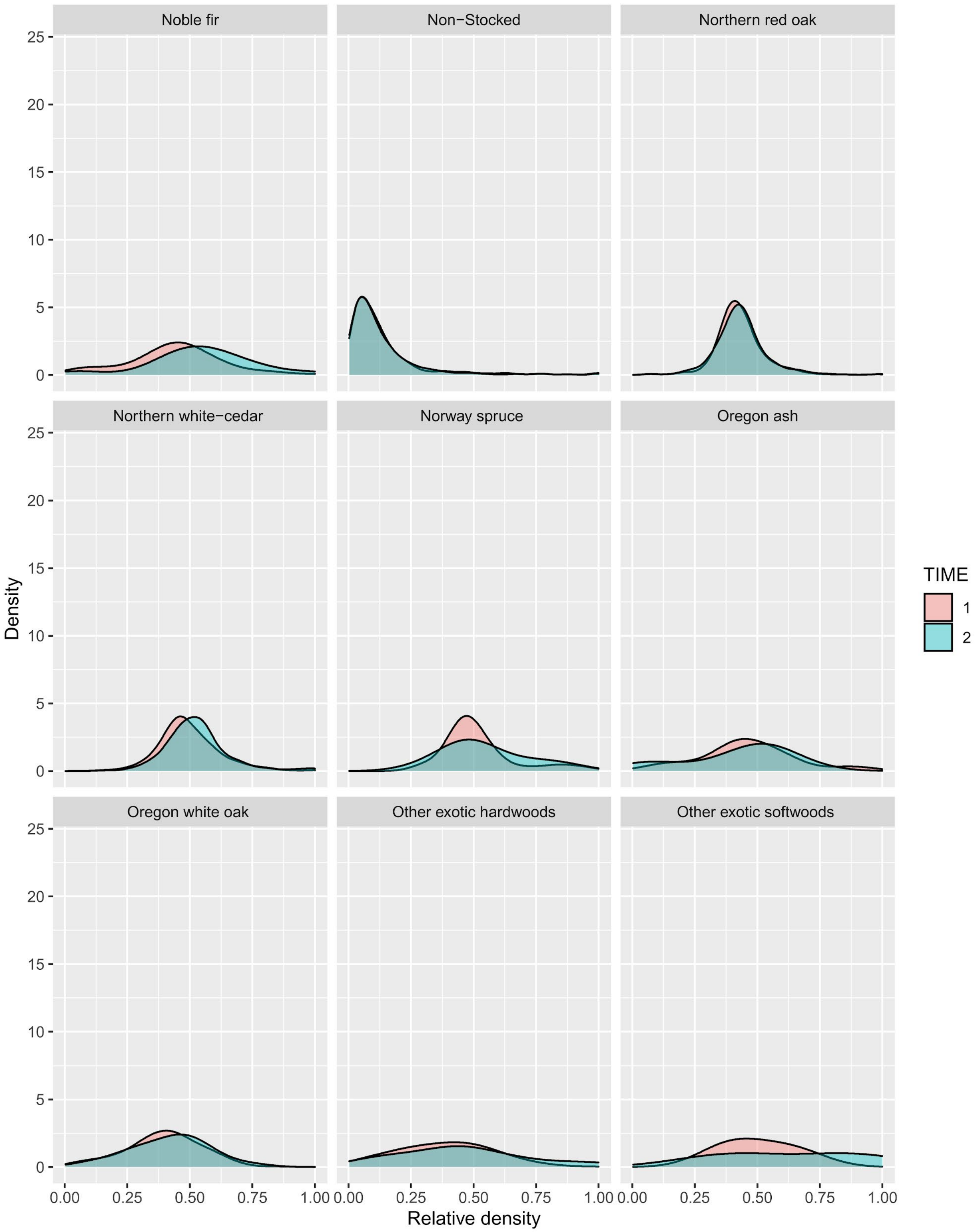
Noble fir
Non−Stocked
Northern red oak
Northern white−cedar
Norway spruce
Oregon ash
Oregon white oak
Other exotic hardwoods
Other exotic softwoods
Density
Relative density
0
5
10
15
20
25
0.00
0.25
0.50
0.75
1.00
TIME
1
2

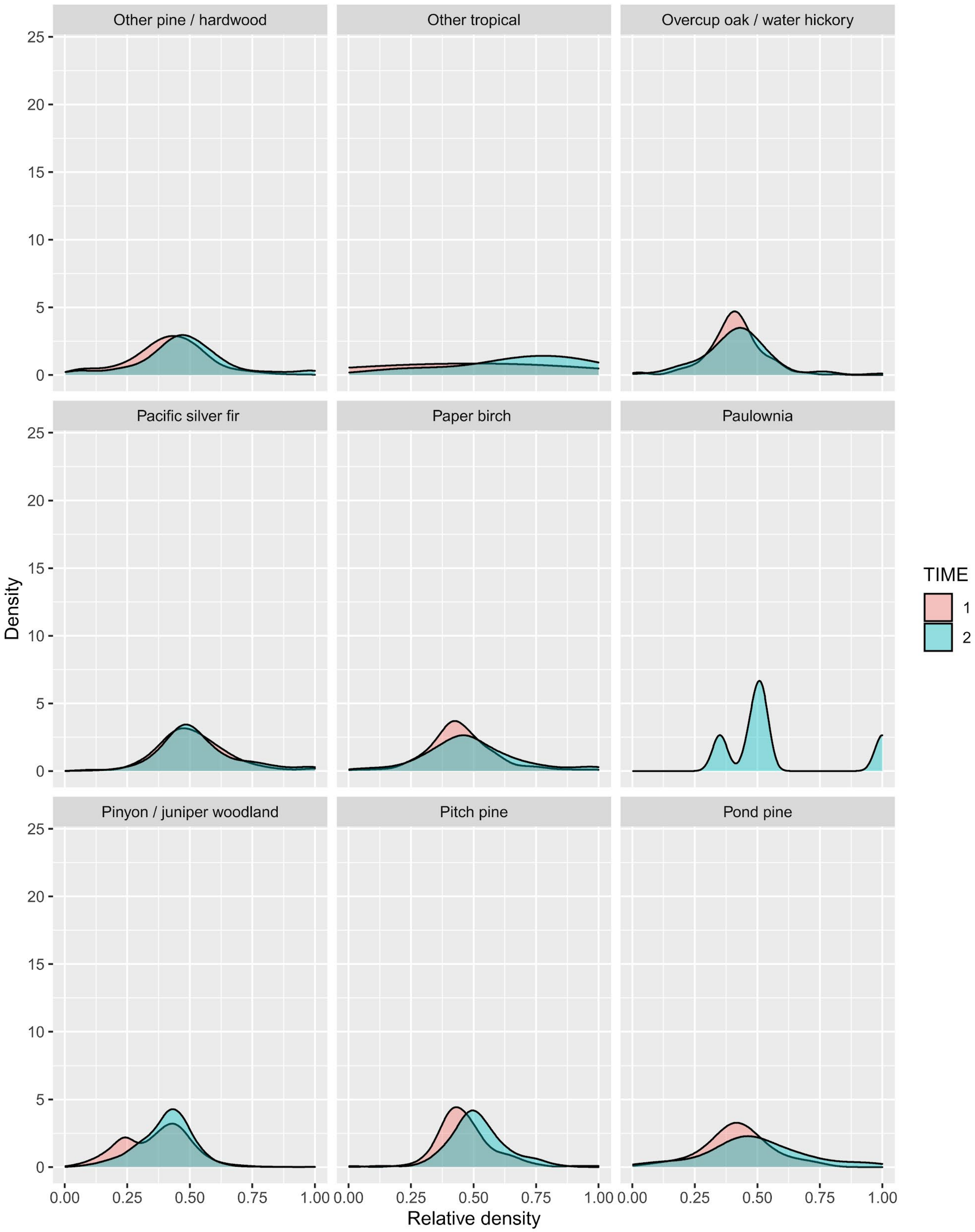
Other pine / hardwood
Other tropical
Overcup oak / water hickory
Pacific silver fir
Paper birch
Paulownia
Pinyon / juniper woodland
Pitch pine
Pond pine
Density
Relative density
TIME
1
2
0
5
10
15
20
25
0.00
0.25
0.50
0.75
1.00

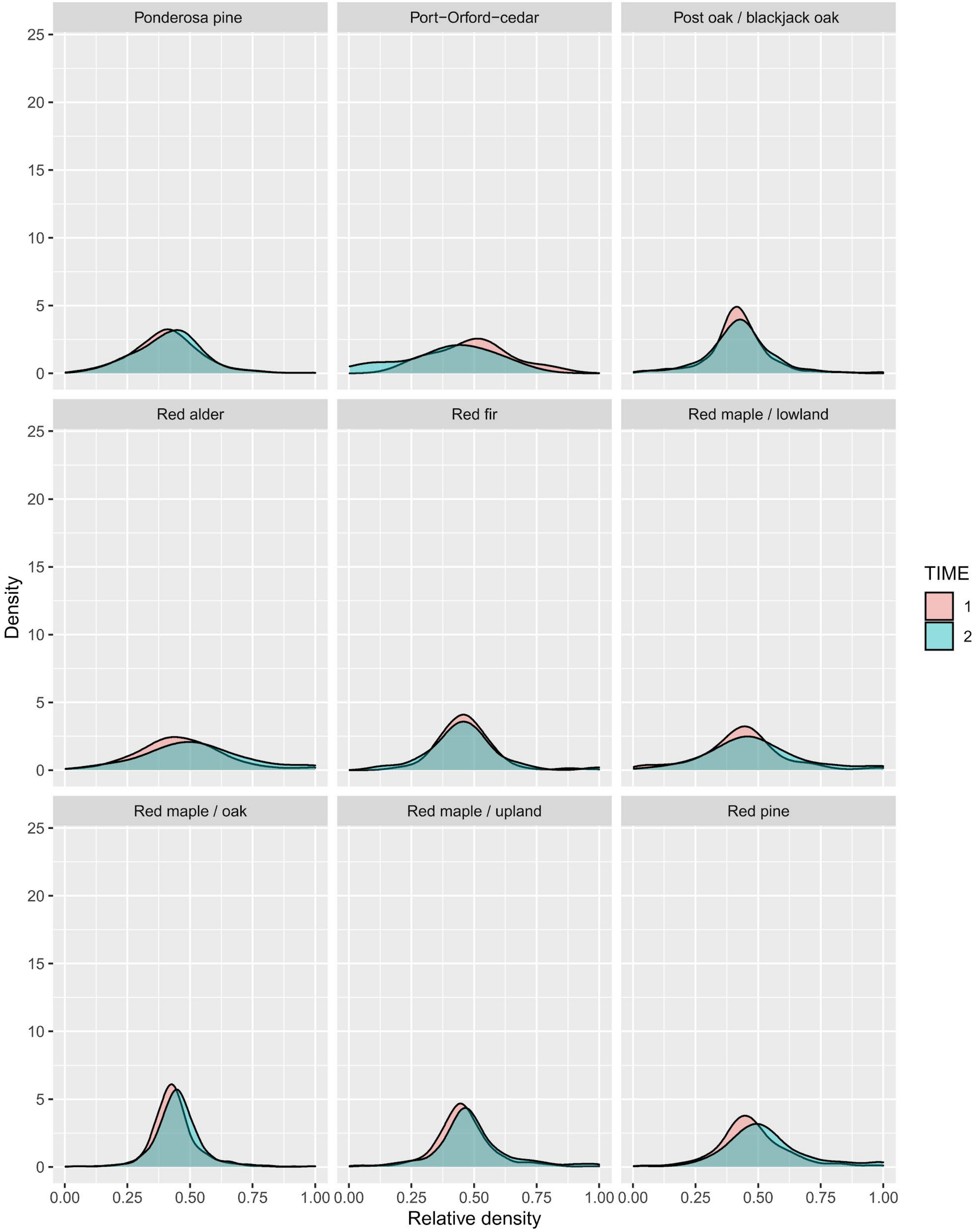
Ponderosa pine
Port−Orford−cedar
Post oak / blackjack oak
Red alder
Red fir
Red maple / lowland
Red maple / oak
Red maple / upland
Red pine
Density
Relative density
TIME
1
2
0
5
10
15
20
25
0.00
0.25
0.50
0.75
1.00

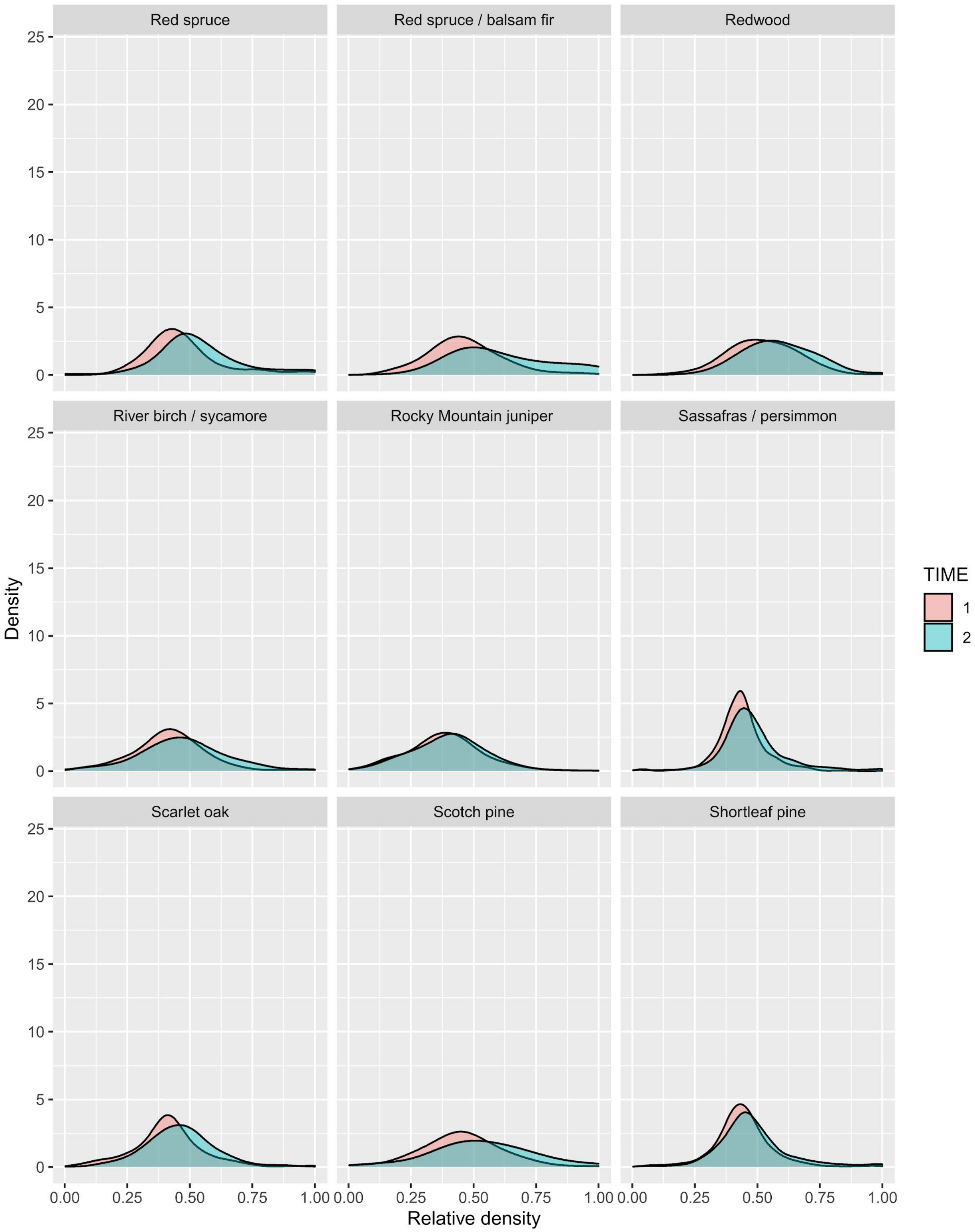
Red spruce
Red spruce / balsam fir
Redwood
River birch / sycamore
Rocky Mountain juniper
Sassafras / persimmon
Scarlet oak
Scotch pine
Shortleaf pine
Density
Relative density
TIME
1
2
0
5
10
15
20
25
0.00
0.25
0.50
0.75
1.00

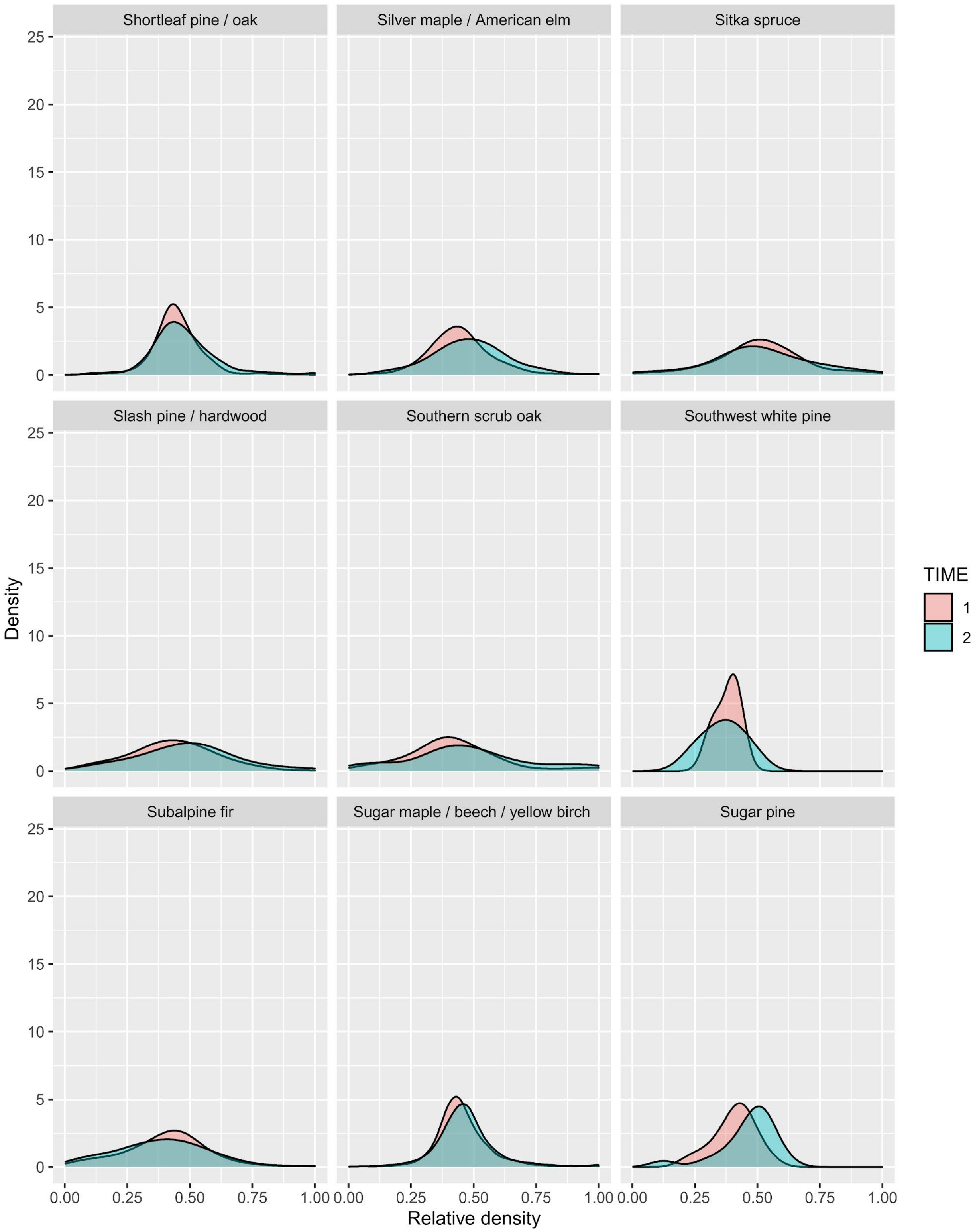
Shortleaf pine / oak
Silver maple / American elm
Sitka spruce
Slash pine / hardwood
Southern scrub oak
Southwest white pine
Subalpine fir
Sugar maple / beech / yellow birch
Sugar pine
Density
Relative density
TIME
1
2
0
5
10
15
20
25
0.00
0.25
0.50
0.75
1.00

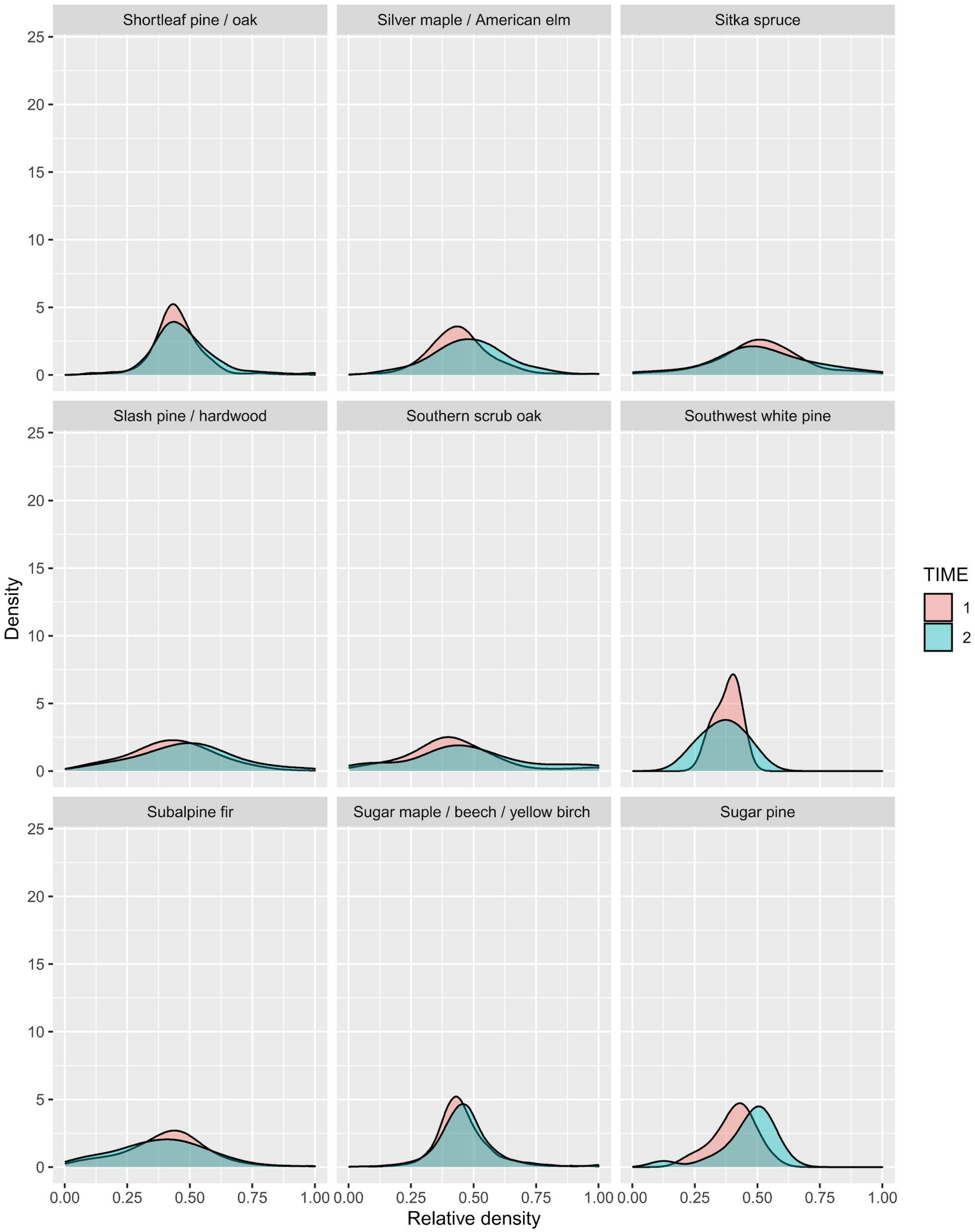
Shortleaf pine / oak
Silver maple / American elm
Sitka spruce
Slash pine / hardwood
Southern scrub oak
Southwest white pine
Subalpine fir
Sugar maple / beech / yellow birch
Sugar pine
Density
Relative density
TIME
1
2
0
5
10
15
20
25
0.00
0.25
0.50
0.75
1.00

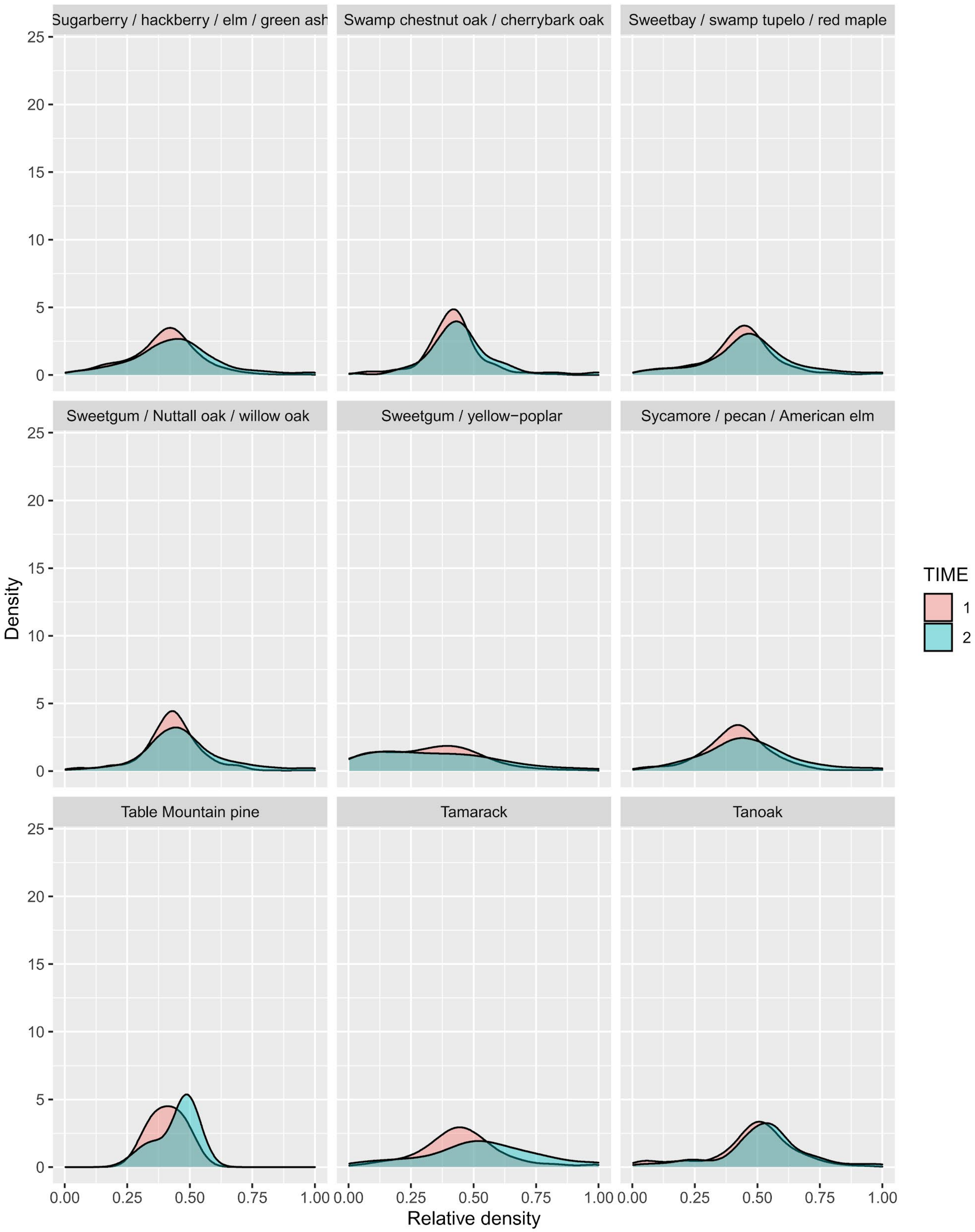

Sugarberry / hackberry / elm / green ash
Swamp chestnut oak / cherrybark oak
Sweetbay / swamp tupelo / red maple
Sweetgum / Nuttall oak / willow oak
Sweetgum / yellow−poplar
Sycamore / pecan / American elm
Table Mountain pine
Tamarack
Tanoak
Density
Relative density
TIME
1
2
0
5
10
15
20
25
0.00
0.25
0.50
0.75
1.00

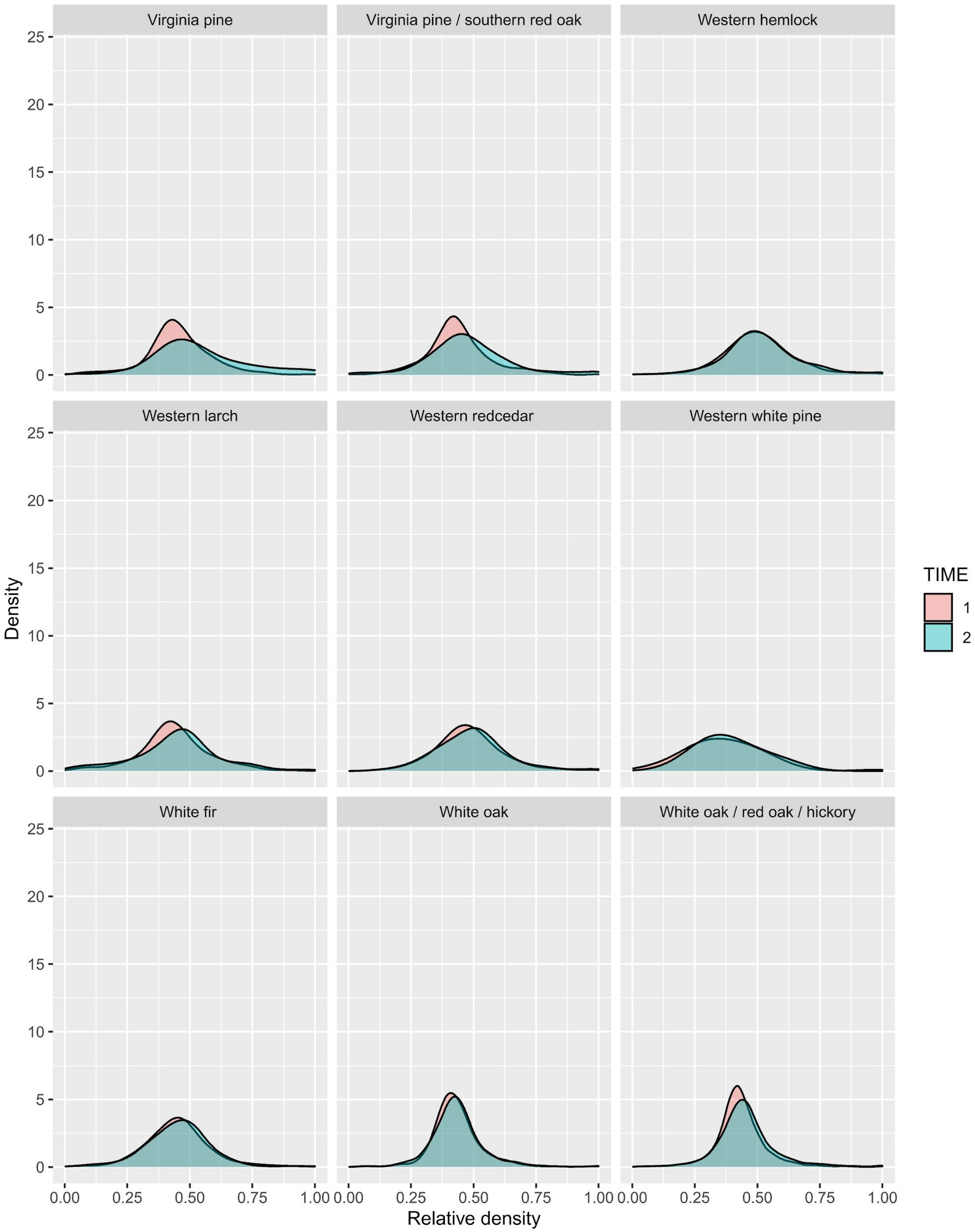
Virginia pine
Virginia pine / southern red oak
Western hemlock
Western larch
Western redcedar
Western white pine
White fir
White oak
White oak / red oak / hickory
Density
0
5
10
15
20
25
0.00
0.25
0.50
0.75
1.00
Relative density
TIME
1
2

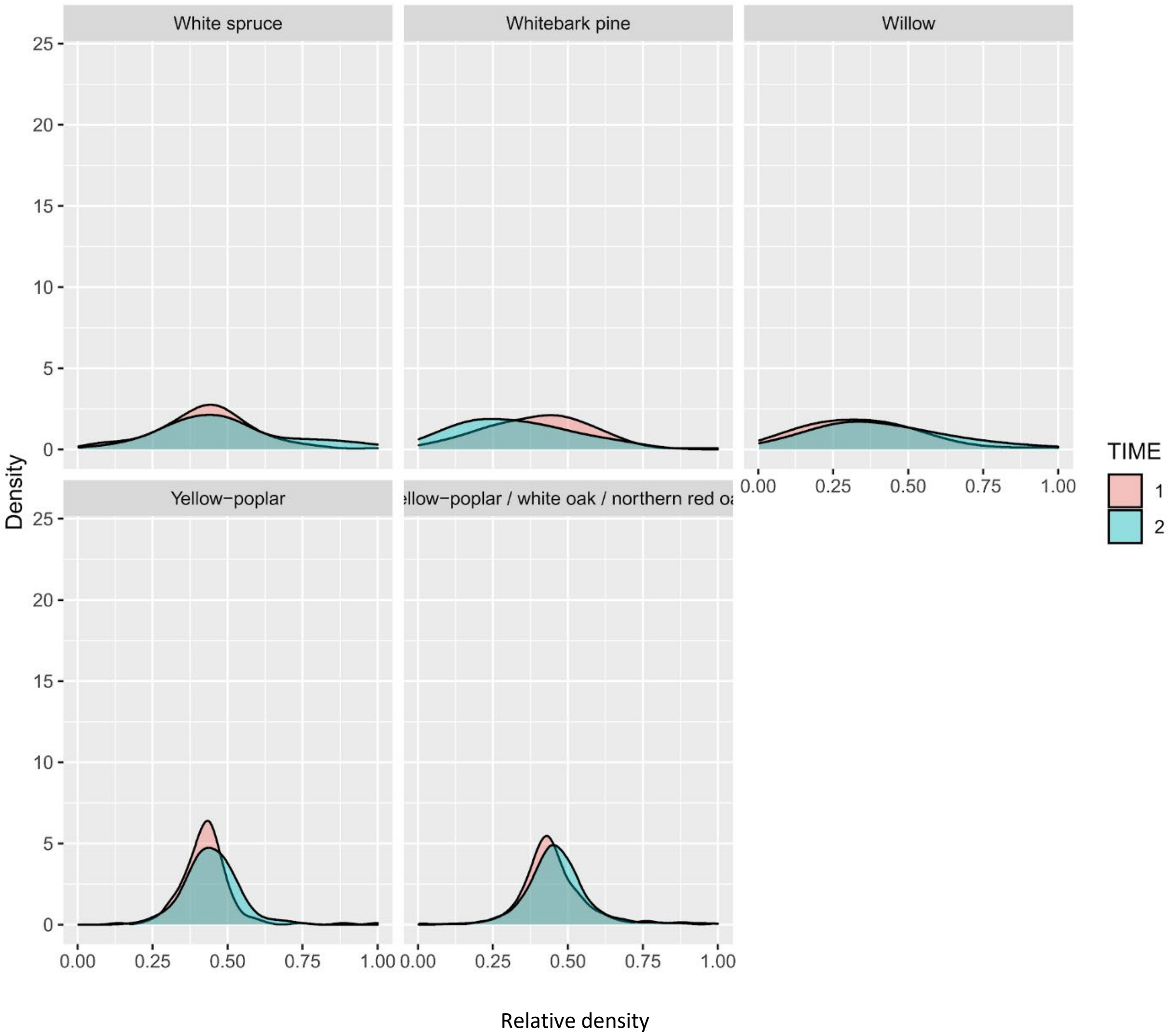

Supplementary Figure 6. Relative frequency distribution of relative density (RD) for Time 1 (1998-2012) and Time 2 (2013-2020) by US Forest Service, Forest Inventory and Analysis (FIA) forest type.

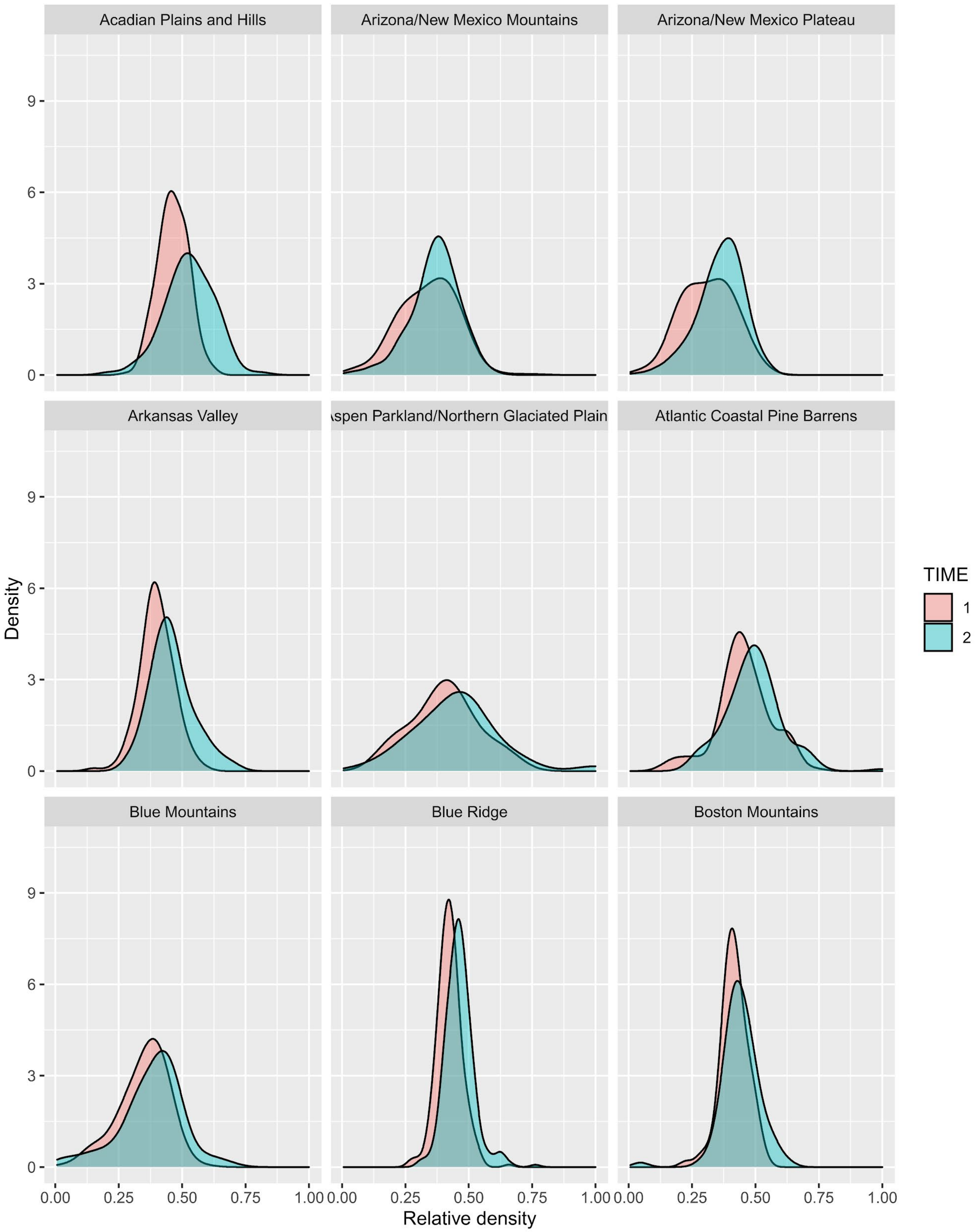
Acadian Plains and Hills
Arizona/New Mexico Mountains
Arizona/New Mexico Plateau
Arkansas Valley
spen Parkland/Northern Glaciated Plain
Atlantic Coastal Pine Barrens
Blue Mountains
Blue Ridge
Boston Mountains
Density
Relative density
TIME
1
2
0
3
6
9
0.00
0.25
0.50
0.75
1.00

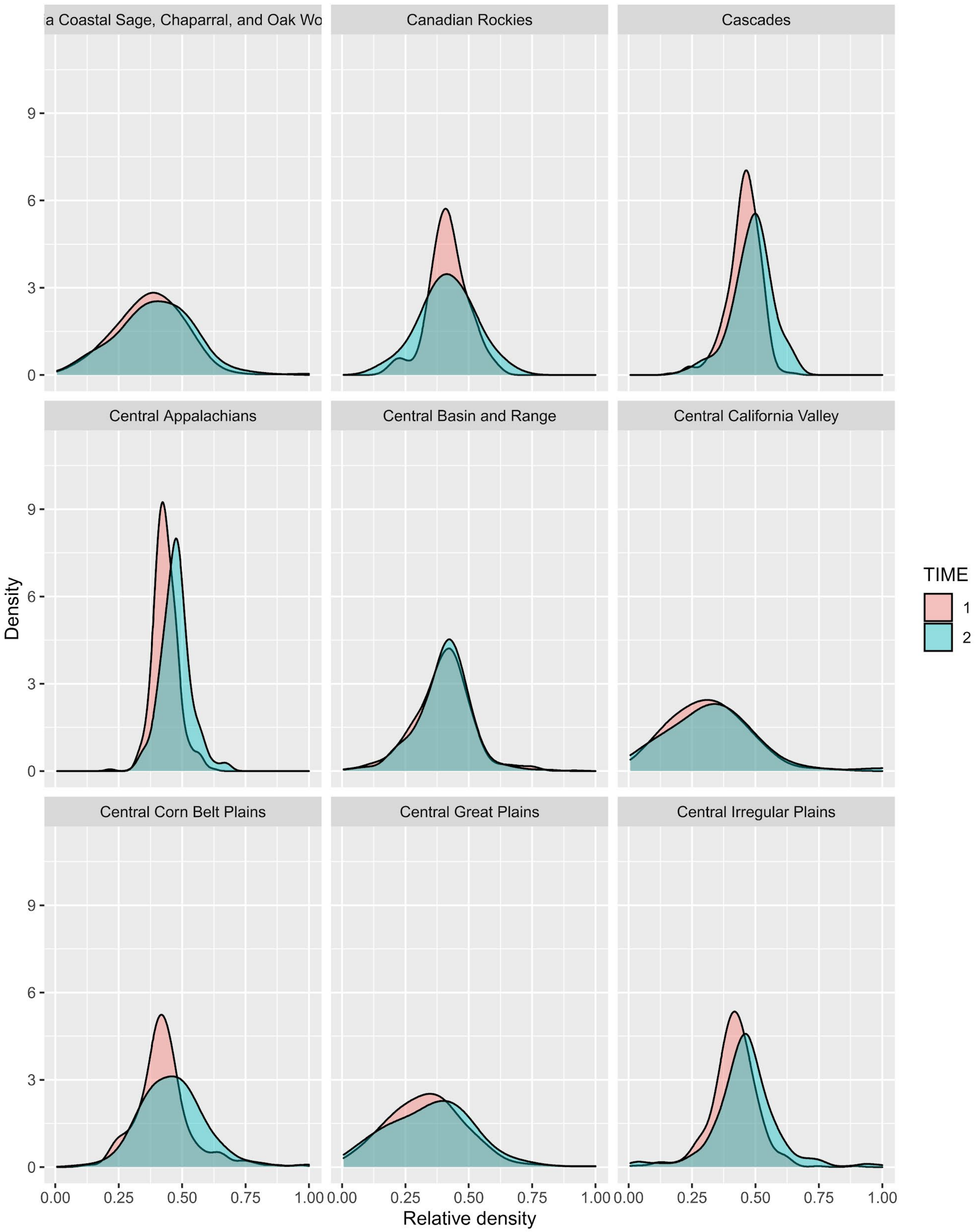

a Coastal Sage, Chaparral, and Oak Wo
Canadian Rockies
Cascades
Central Appalachians
Central Basin and Range
Central California Valley
Central Corn Belt Plains
Central Great Plains
Central Irregular Plains
Density
9
6
3
0
0.00
0.25
0.50
0.75
1.00
Relative density
TIME
1
2

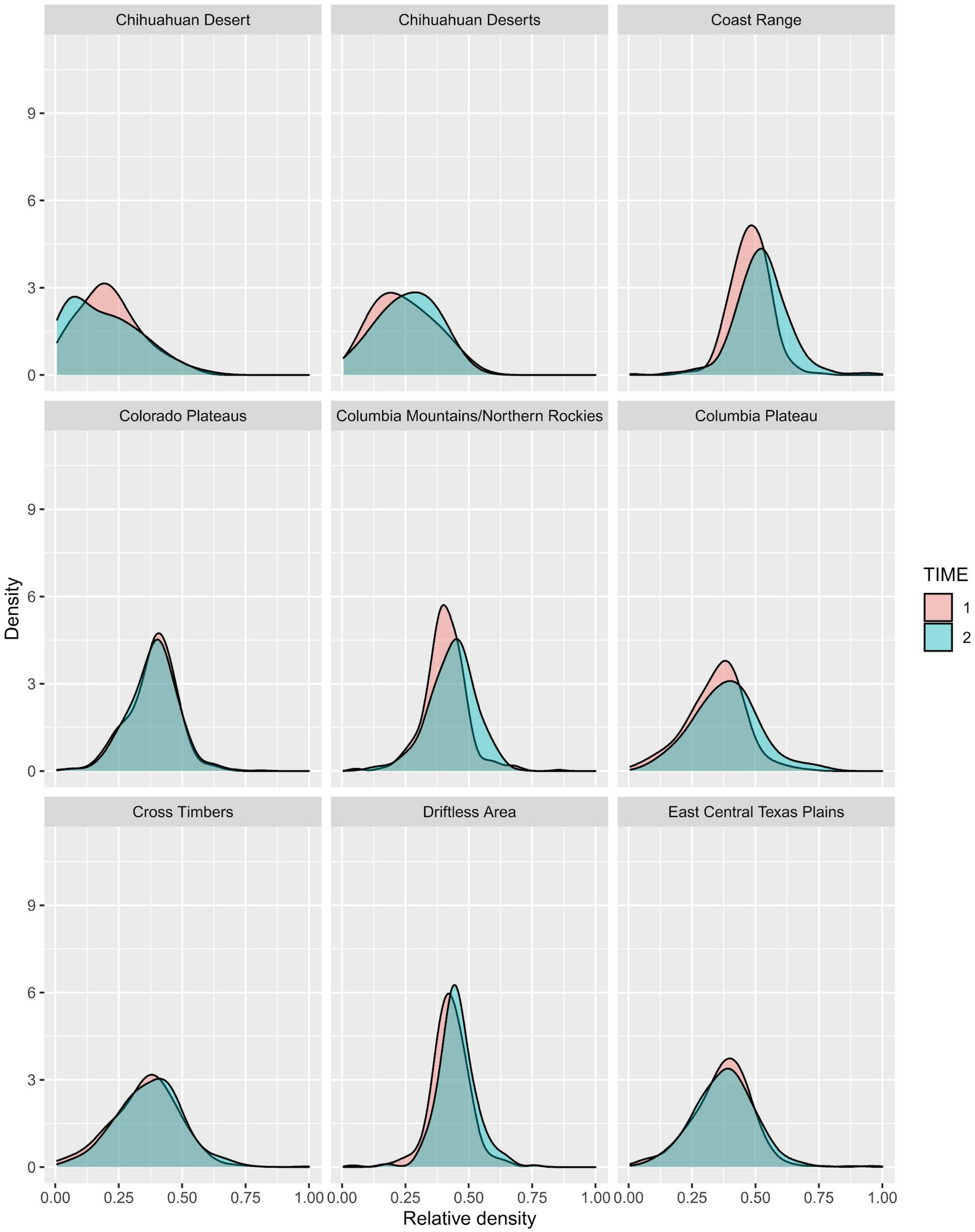
Chihuahuan Desert
Chihuahuan Deserts
Coast Range
Colorado Plateaus
Columbia Mountains/Northern Rockies
Columbia Plateau
Cross Timbers
Driftless Area
East Central Texas Plains
Density
0
3
6
9
Relative density
0.00
0.25
0.50
0.75
1.00
TIME
1
2

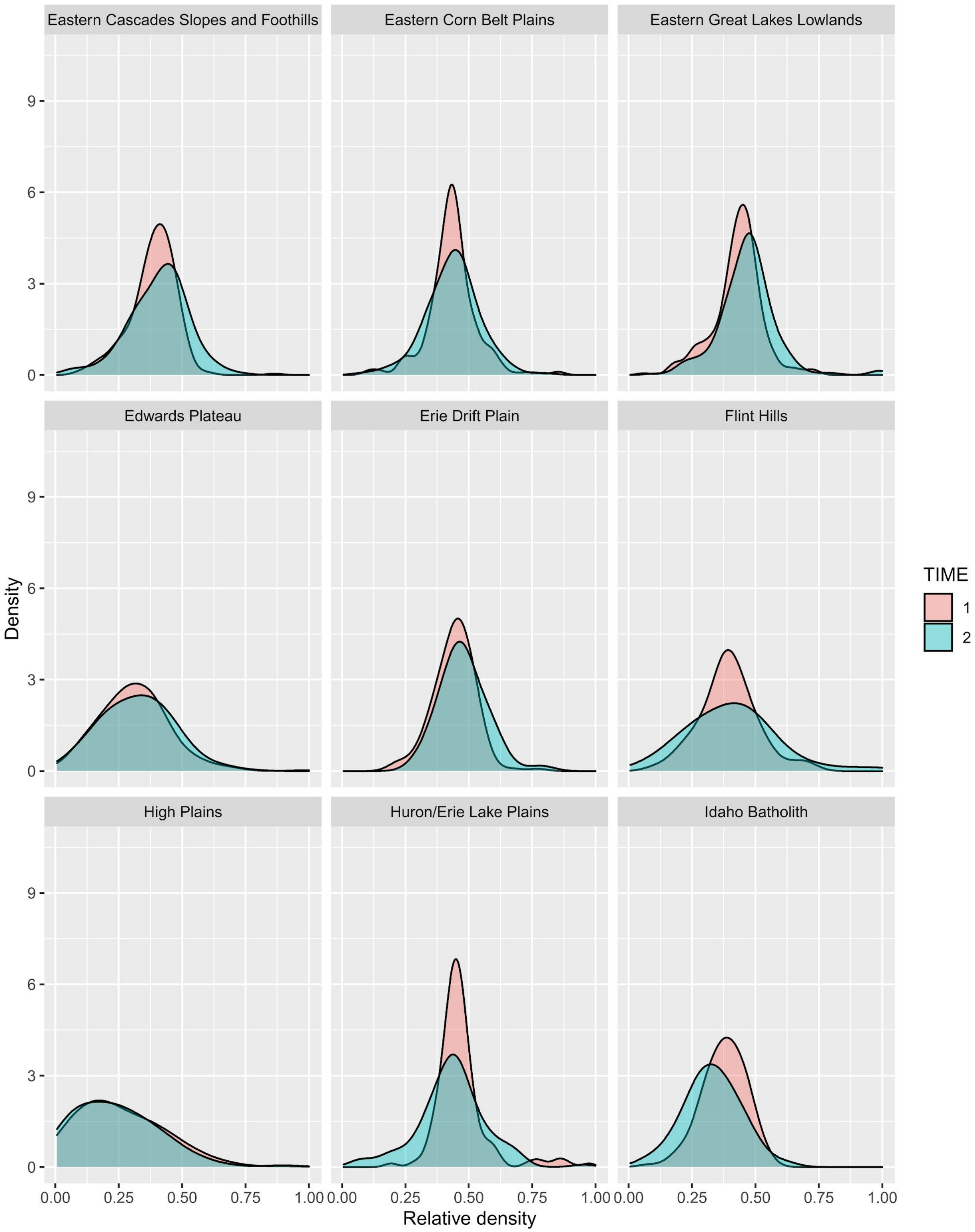
Eastern Cascades Slopes and Foothills
Eastern Corn Belt Plains
Eastern Great Lakes Lowlands
Edwards Plateau
Erie Drift Plain
Flint Hills
High Plains
Huron/Erie Lake Plains
Idaho Batholith
Density
Relative density
TIME
1
2

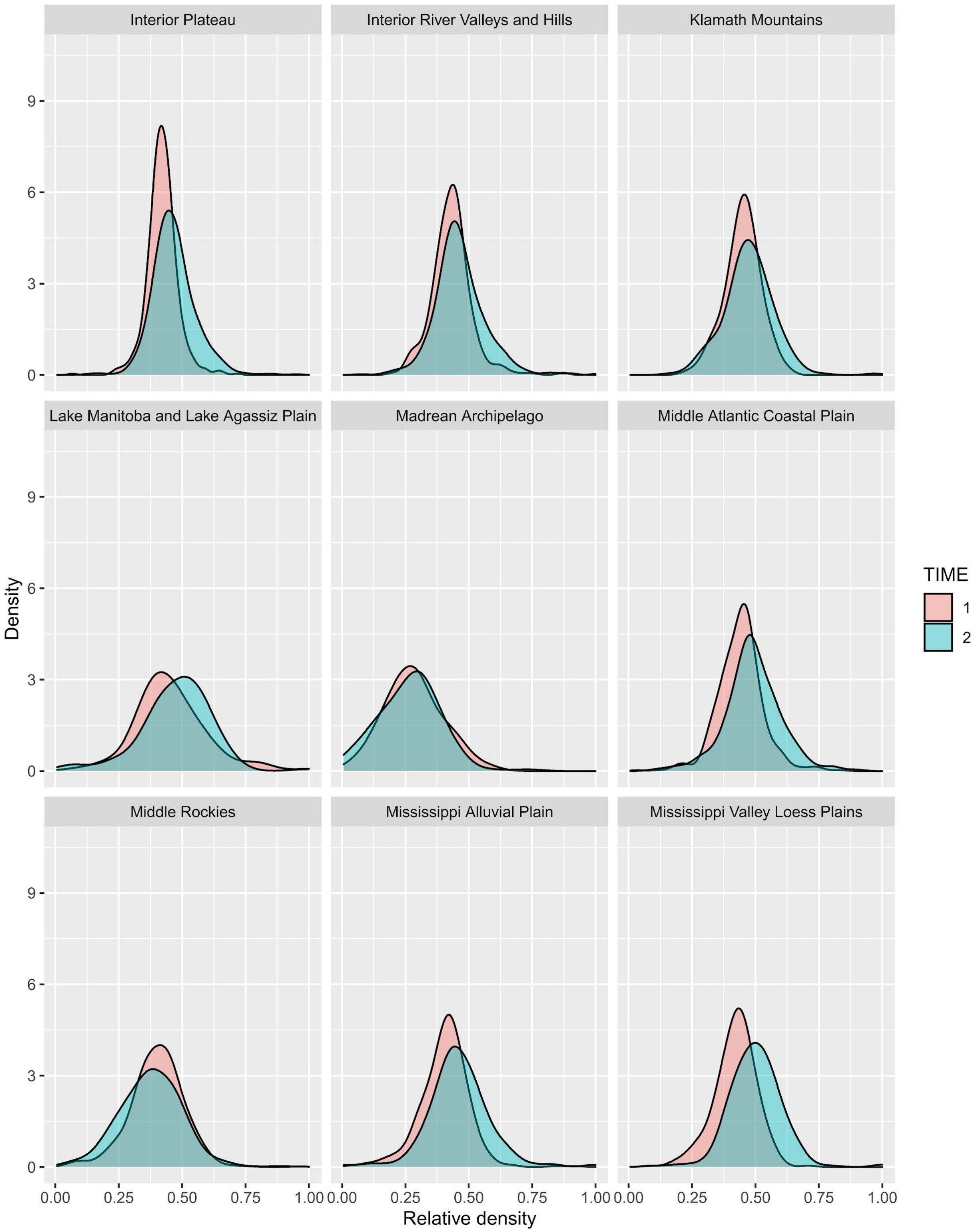
Interior Plateau
Interior River Valleys and Hills
Klamath Mountains
Lake Manitoba and Lake Agassiz Plain
Madrean Archipelago
Middle Atlantic Coastal Plain
Middle Rockies
Mississippi Alluvial Plain
Mississippi Valley Loess Plains
Density
Relative density
TIME
1
2
0
3
6
9
0.00
0.25
0.50
0.75
1.00

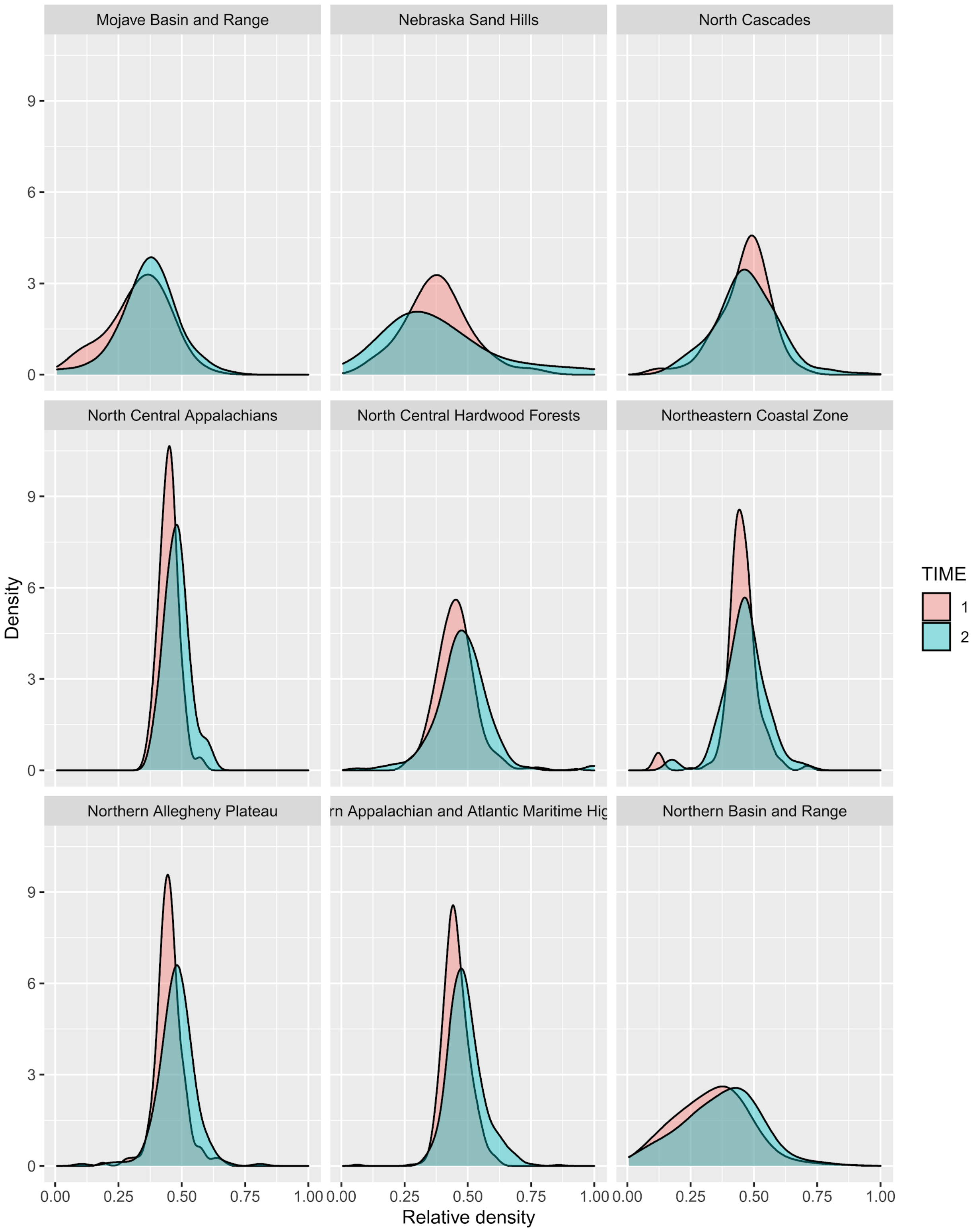
Mojave Basin and Range
Nebraska Sand Hills
North Cascades
North Central Appalachians
North Central Hardwood Forests
Northeastern Coastal Zone
Northern Allegheny Plateau
rn Appalachian and Atlantic Maritime Hig
Northern Basin and Range
Density
Relative density
TIME
1
2
0
3
6
9
0.00
0.25
0.50
0.75
1.00

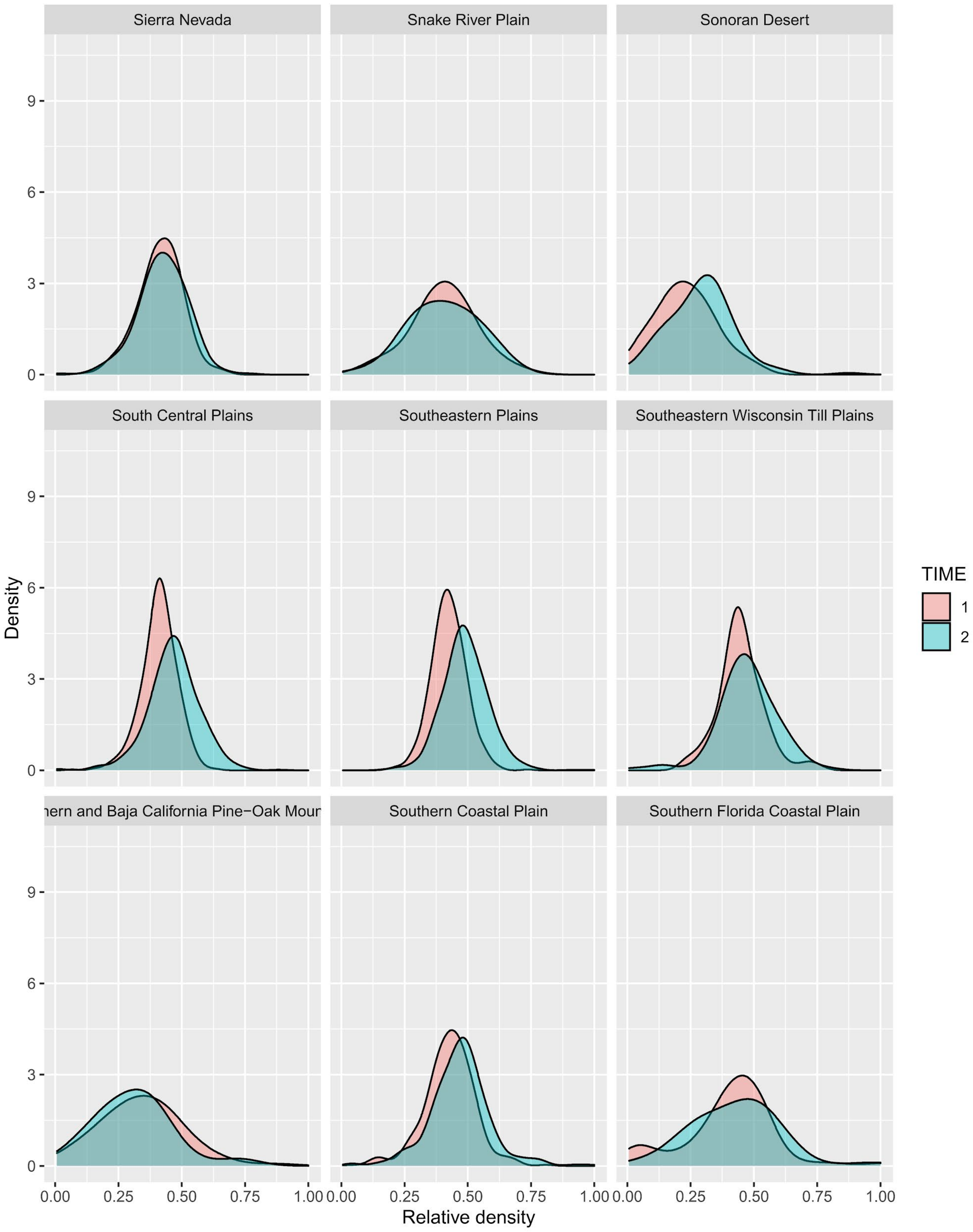
Sierra Nevada
Snake River Plain
Sonoran Desert
South Central Plains
Southeastern Plains
Southeastern Wisconsin Till Plains
hern and Baja California Pine−Oak Moun
Southern Coastal Plain
Southern Florida Coastal Plain
Density
0
3
6
9
0.00
0.25
0.50
0.75
1.00
Relative density
TIME
1
2

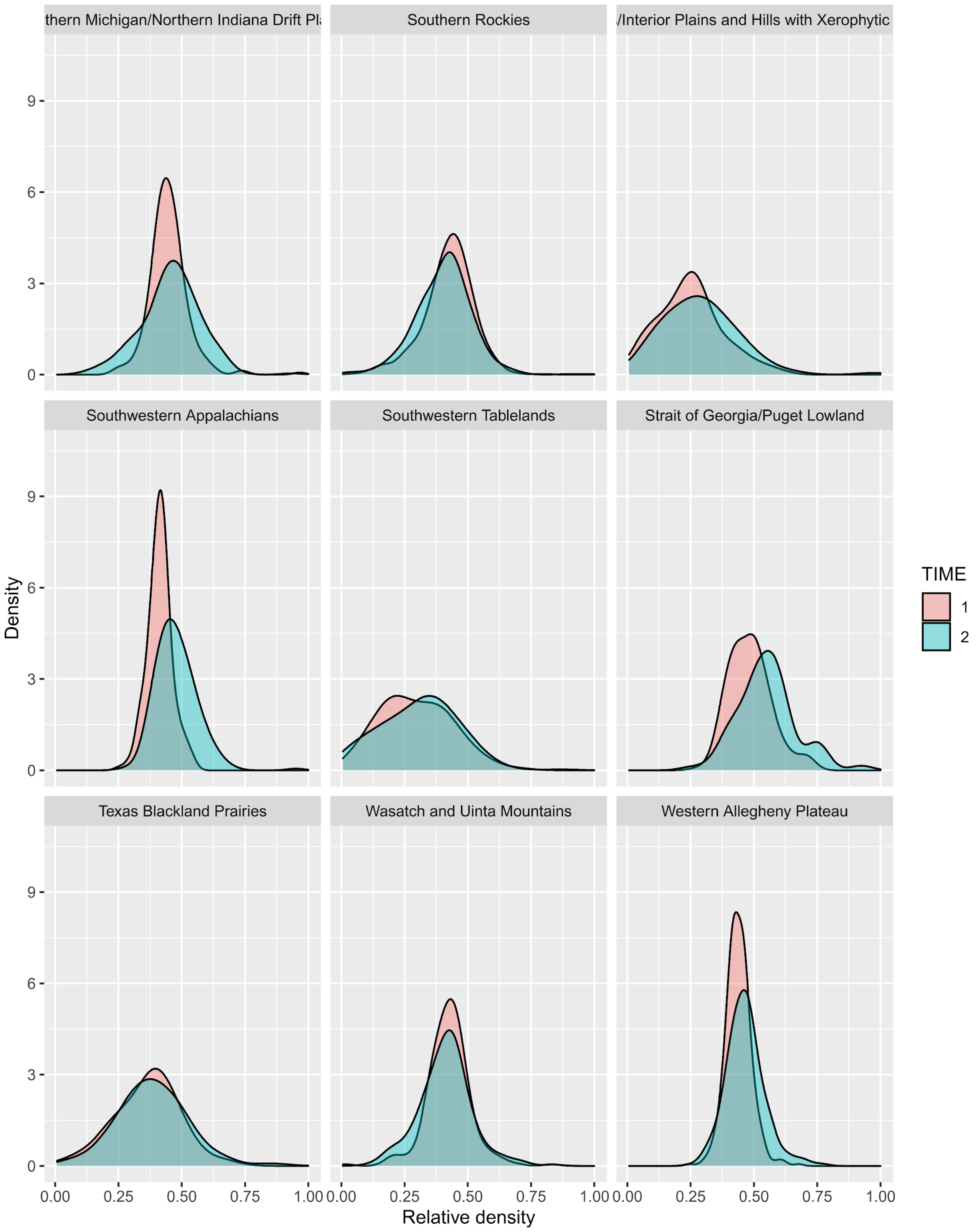

thern Michigan/Northern Indiana Drift Pla
Southern Rockies
/Interior Plains and Hills with Xerophytic
Southwestern Appalachians
Southwestern Tablelands
Strait of Georgia/Puget Lowland
Texas Blackland Prairies
Wasatch and Uinta Mountains
Western Allegheny Plateau
Density
Relative density
TIME
1
2
0
3
6
9
0.00
0.25
0.50
0.75
1.00

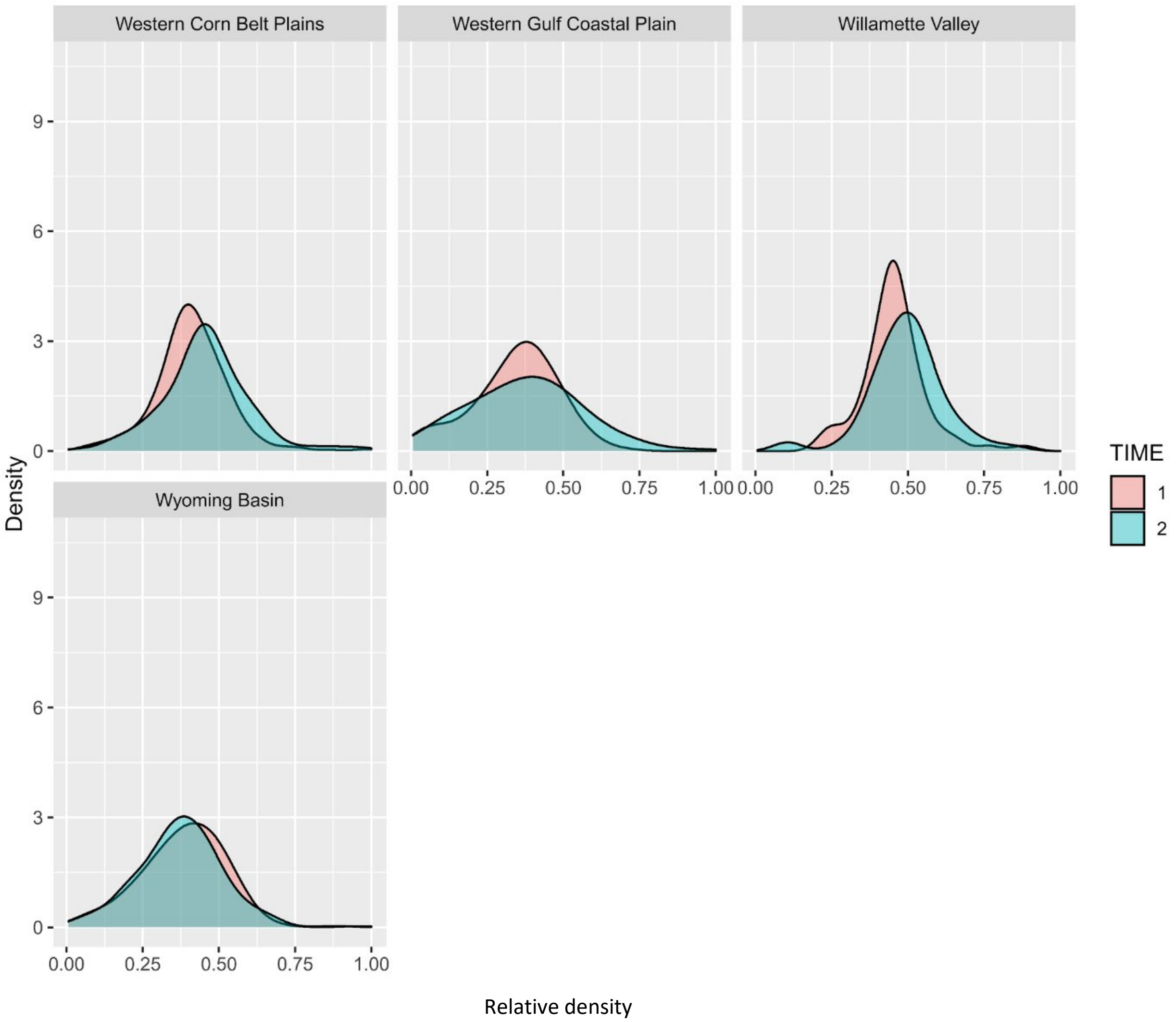

Supplementary Figure 7. Relative frequency distribution of relative density (RD) for Time 1 (1999-2012) and Time 2 (2013-2020) by US EPA Level III ecoregion.

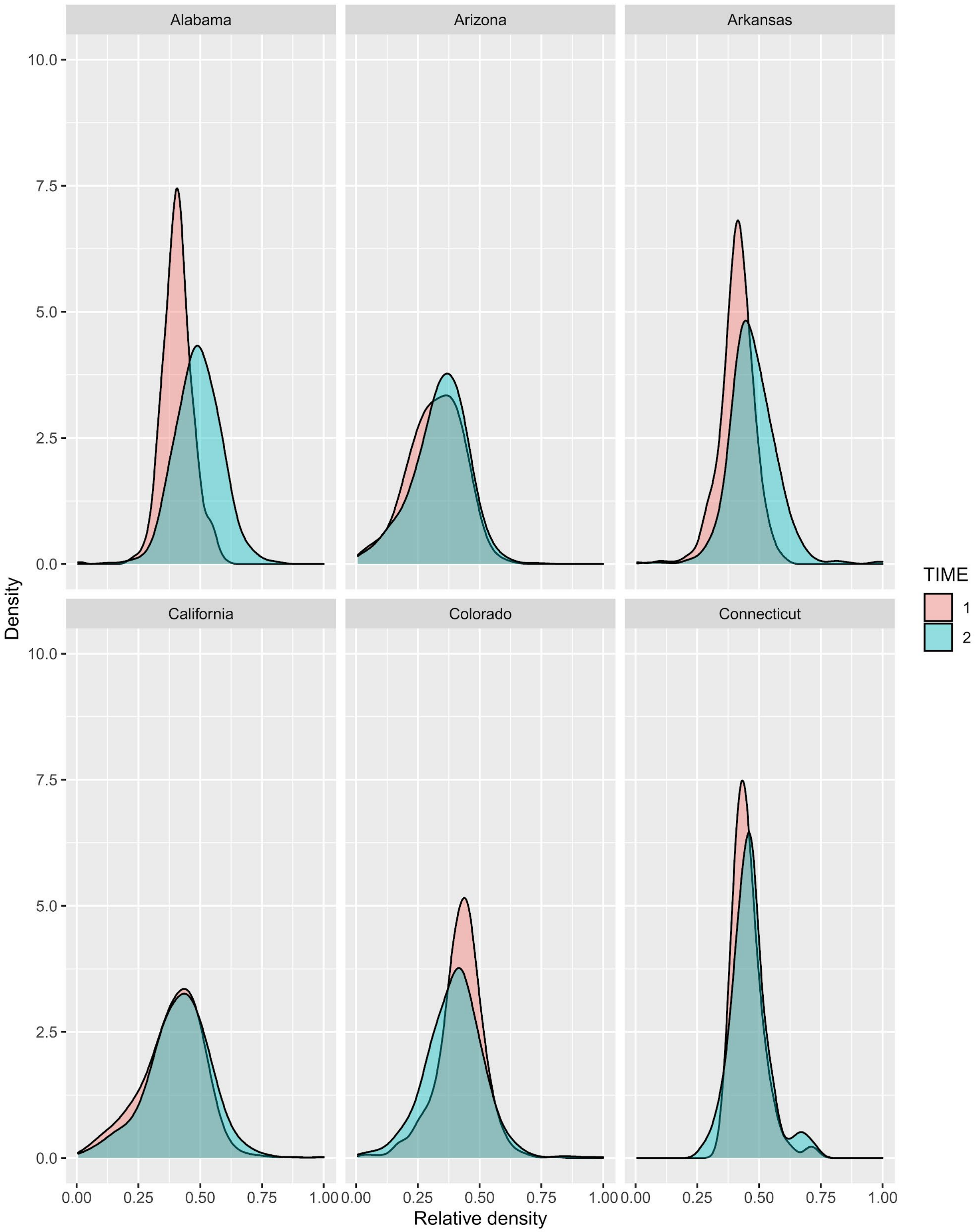
Alabama
Arizona
Arkansas
California
Colorado
Connecticut
Density
10.0
7.5
5.0
2.5
0.0
0.00
0.25
0.50
0.75
1.00
Relative density
TIME
1
2

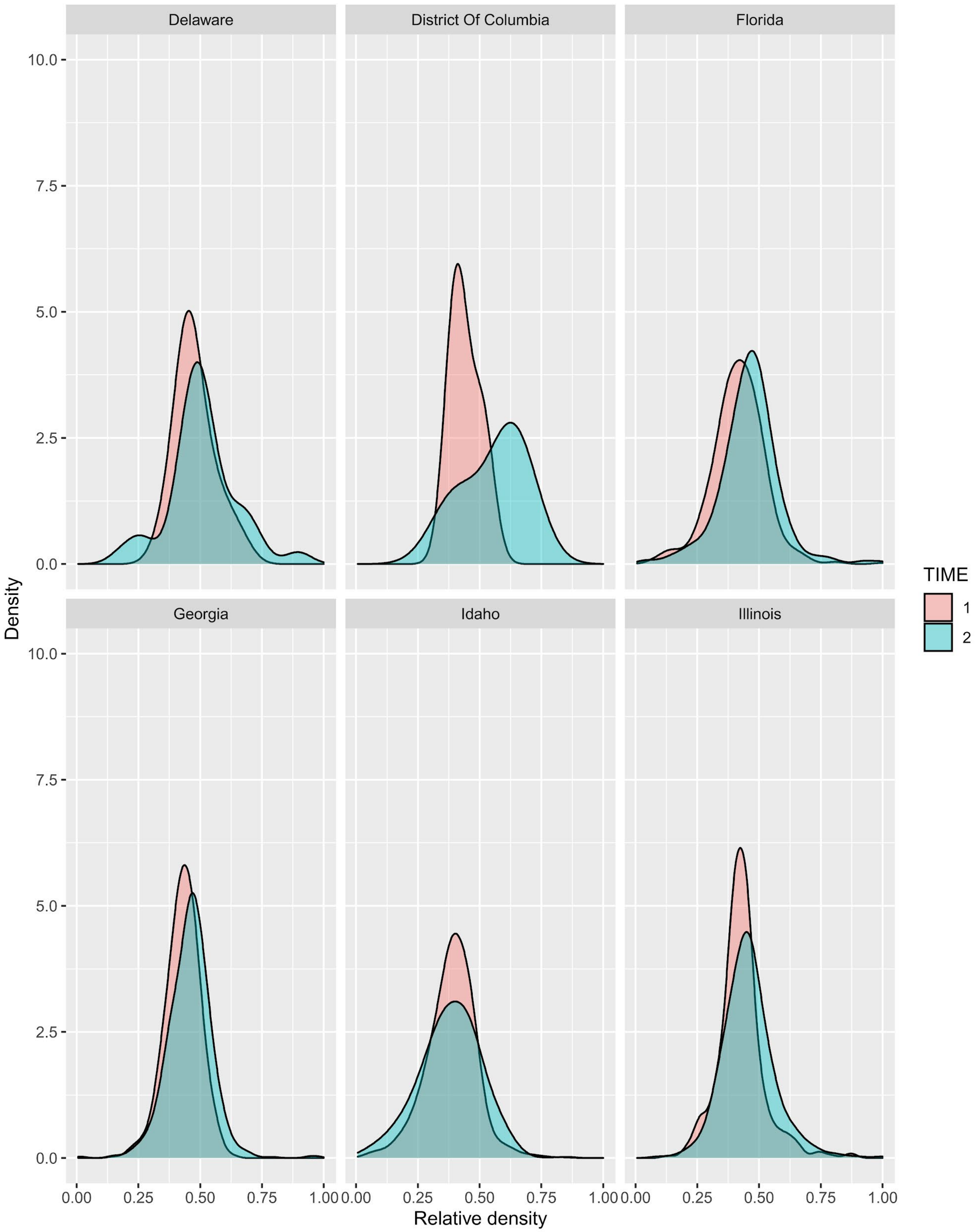
Delaware
District Of Columbia
Florida
Georgia
Idaho
Illinois
Density
Relative density
TIME
1
2
0.0
2.5
5.0
7.5
10.0
0.00
0.25
0.50
0.75
1.00

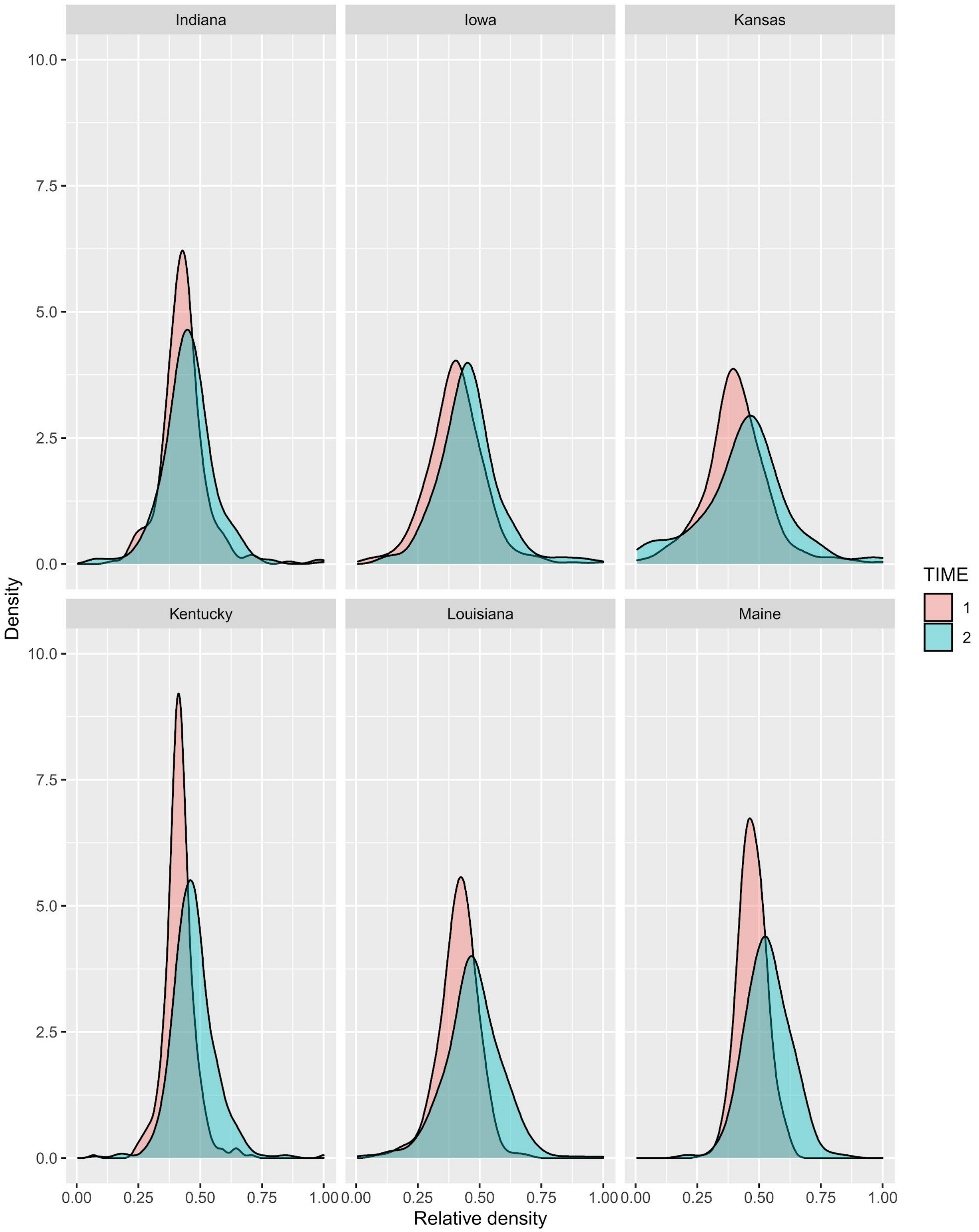

Indiana
Iowa
Kansas
Kentucky
Louisiana
Maine
Density
Relative density
TIME
1
2
0.0
2.5
5.0
7.5
10.0
0.00
0.25
0.50
0.75
1.00

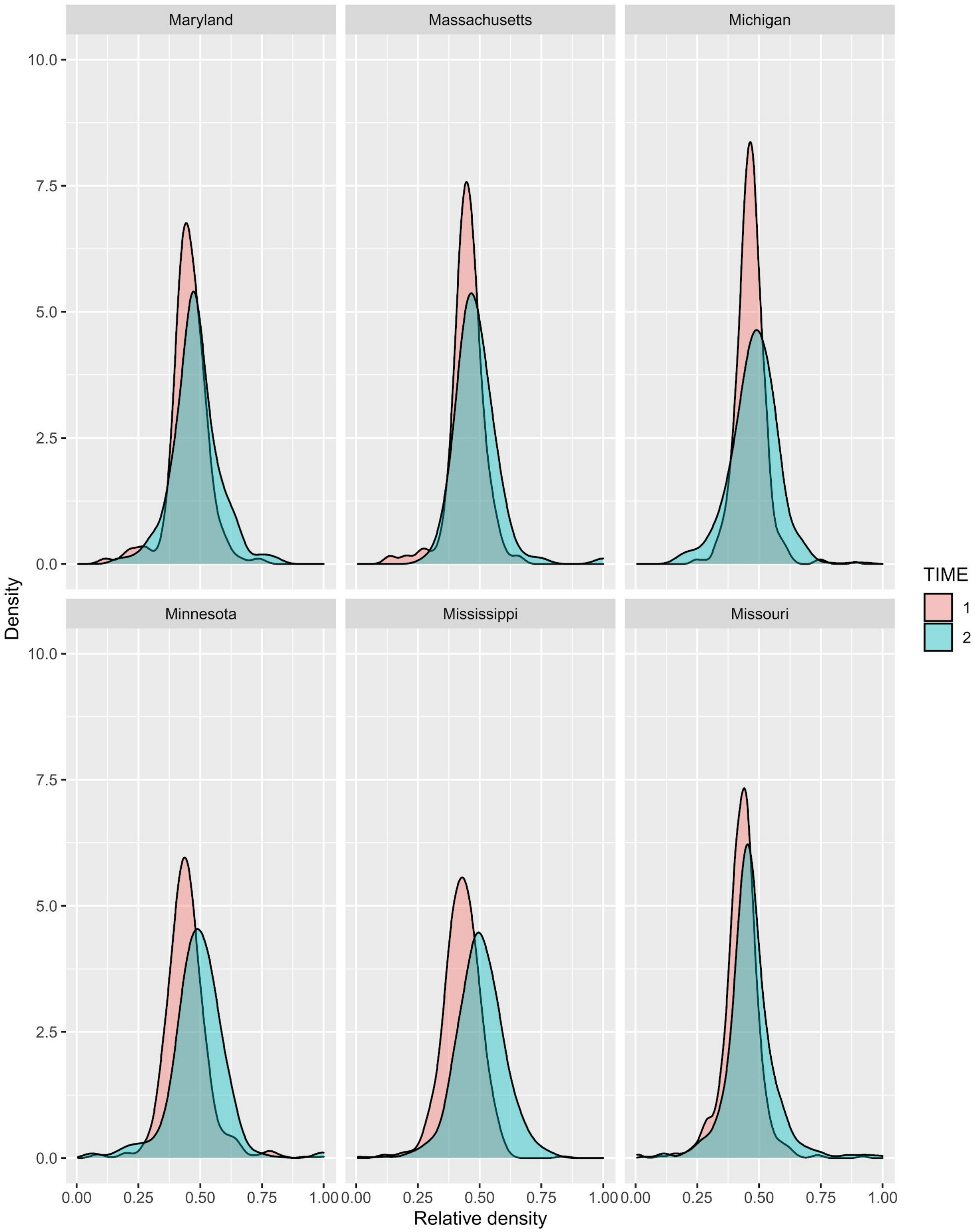
Maryland
Massachusetts
Michigan
Minnesota
Mississippi
Missouri
Density
Relative density
TIME
1
2
0.0
2.5
5.0
7.5
10.0
0.00
0.25
0.50
0.75
1.00

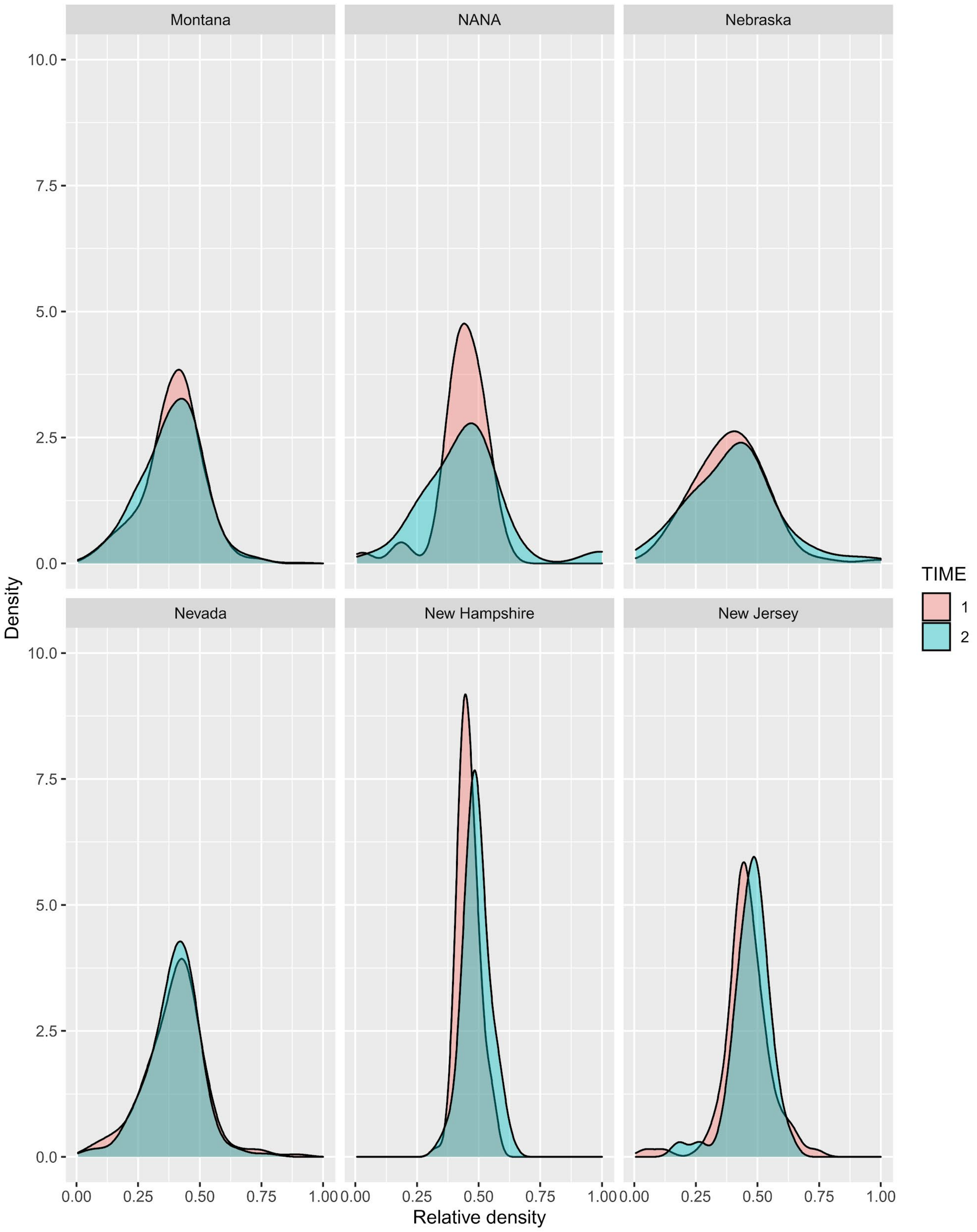

Montana
NANA
Nebraska
Nevada
New Hampshire
New Jersey
10.0
7.5
5.0
2.5
0.0
Density
0.00
0.25
0.50
0.75
1.00
Relative density
TIME
1
2

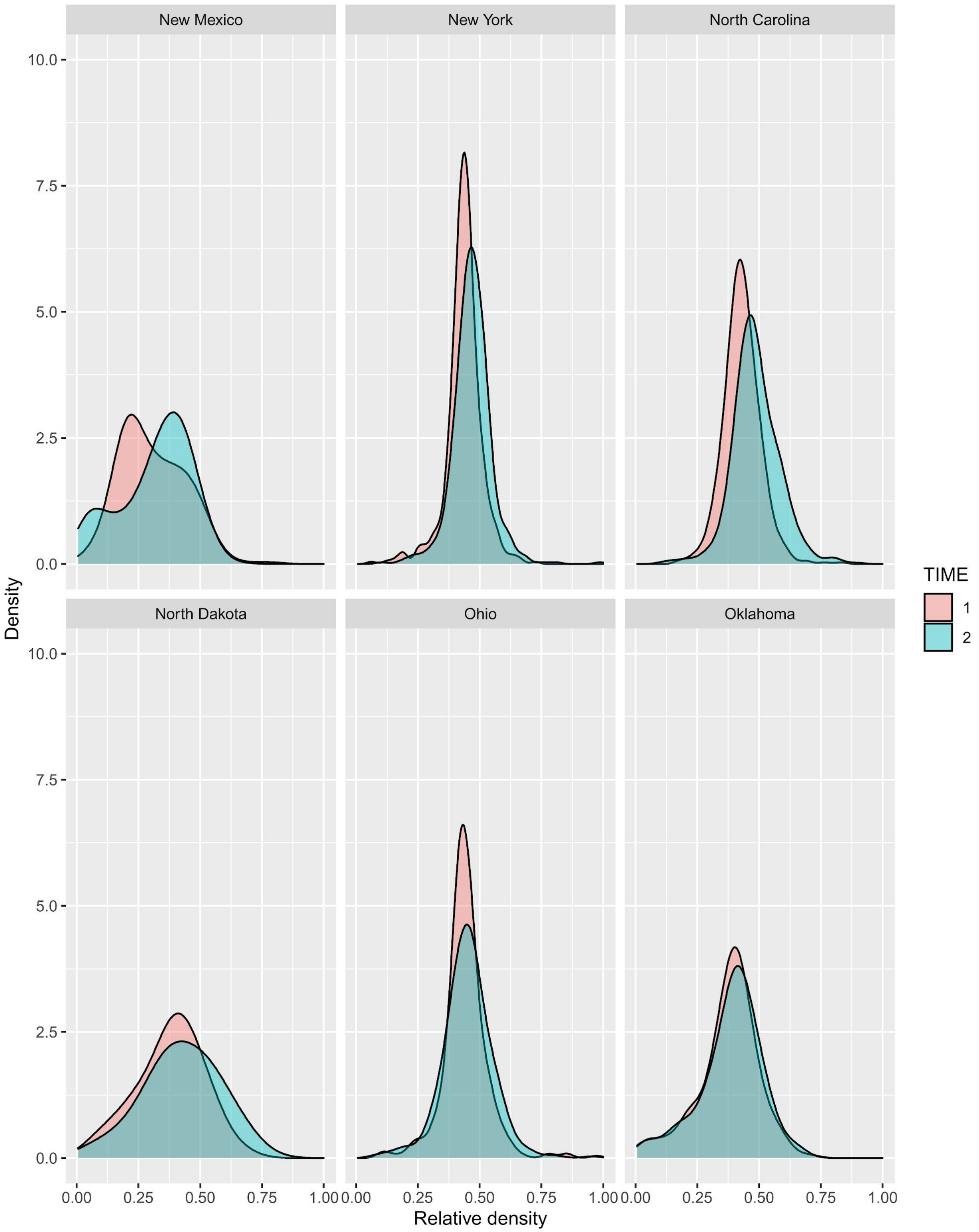
New Mexico
New York
North Carolina
North Dakota
Ohio
Oklahoma
Density
10.0
7.5
5.0
2.5
0.0
0.00
0.25
0.50
0.75
1.00
Relative density
TIME
1
2

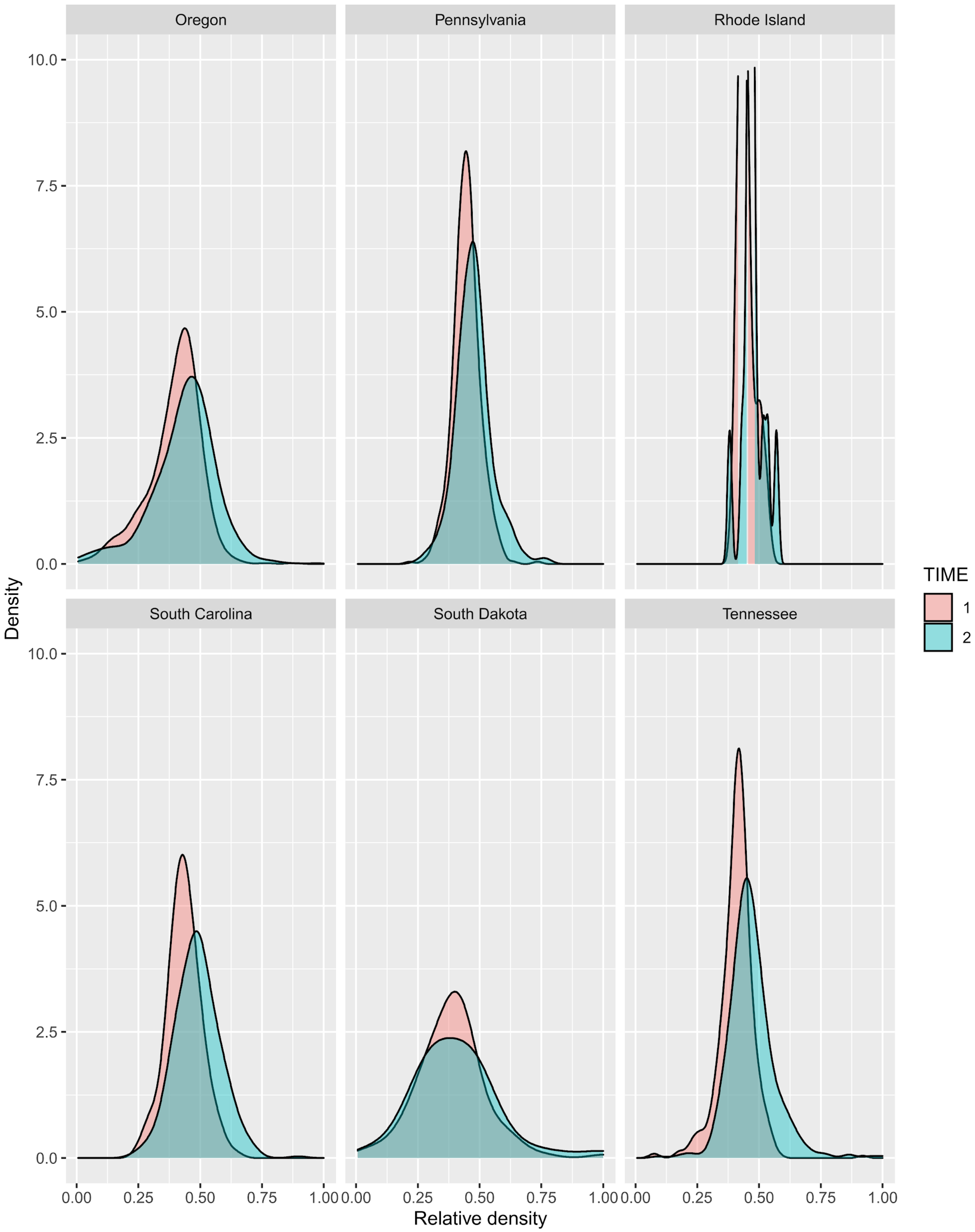
Oregon
Pennsylvania
Rhode Island
South Carolina
South Dakota
Tennessee
Density
Relative density
TIME
1
2
0.0
2.5
5.0
7.5
10.0
0.00
0.25
0.50
0.75
1.00

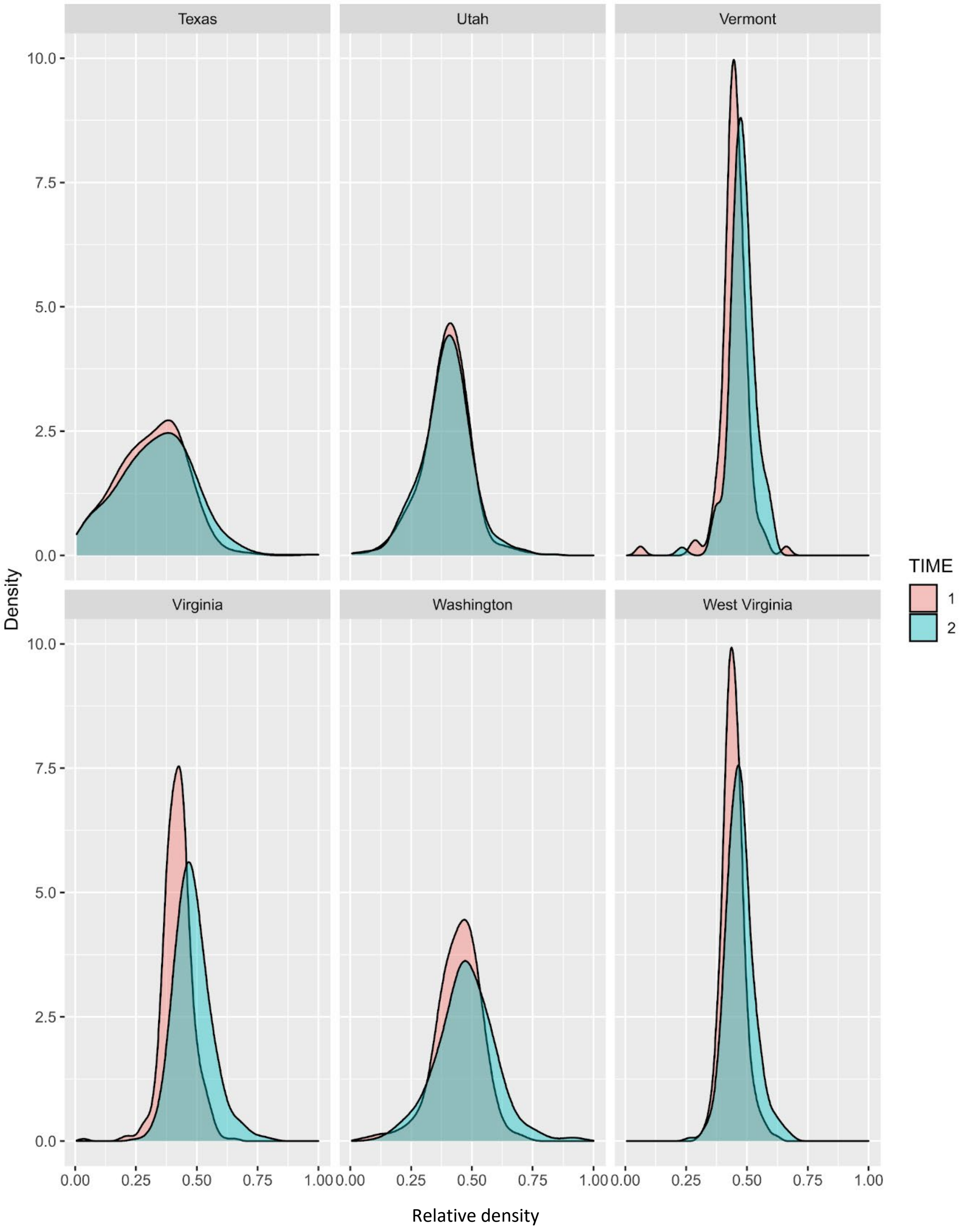

Supplementary Figure 8. Relative frequency distribution of relative density (RD) for Time 1 (1999-2012) and Time 2 (2013-2020) by US state.
